# Molecular Epidemiology and Genetic Evolution of the Whole Genome of G3P[8] Human Rotavirus in Wuhan, China, from 2000 through 2013

**DOI:** 10.1371/journal.pone.0088850

**Published:** 2014-03-27

**Authors:** Yuan-Hong Wang, Bei-Bei Pang, Souvik Ghosh, Xuan Zhou, Tsuzumi Shintani, Noriko Urushibara, Yu-Wei Song, Ming-Yang He, Man-Qing Liu, Wei-Feng Tang, Jin-Song Peng, Quan Hu, Dun-Jin Zhou, Nobumichi Kobayashi

**Affiliations:** 1 Virology section, Wuhan Centers for Disease Prevention and Control, Wuhan, Hubei Province, P.R. China; 2 Department of Hygiene, Sapporo Medical University School of Medicine, Sapporo, Japan; 3 College of Life Sciences, Central China Normal University, Wuhan, Hubei Province, P.R. China; University of Hong Kong, Hong Kong

## Abstract

**Background:**

Rotaviruses are a major etiologic agent of gastroenteritis in infants and young children worldwide. Since the latter of the 1990s, G3 human rotaviruses referred to as “new variant G3” have emerged and spread in China, being a dominant genotype until 2010, although their genomic evolution has not yet been well investigated.

**Methods:**

The complete genomes of 33 G3P[8] human rotavirus strains detected in Wuhan, China, from 2000 through 2013 were analyzed. Phylogenetic trees of concatenated sequences of all the RNA segments and individual genes were constructed together with published rotavirus sequences.

**Results:**

Genotypes of 11 gene segments of all the 33 strains were assigned to G3-P[8]-I1-R1-C1-M1-A1-N1-T1-E1-H1, belonging to Wa genogroup. Phylogenetic analysis of the concatenated full genome sequences indicated that all the modern G3P[8] strains were assigned to Cluster 2 containing only one clade of G3P[8] strains in the US detected in the 1970s, which was distinct from Cluster 1 comprising most of old G3P[8] strains. While main lineages of all the 11 gene segments persisted during the study period, different lineages appeared occasionally in RNA segments encoding VP1, VP4, VP6, and NSP1-NSP5, exhibiting various allele constellations. In contrast, only a single lineage was detected for VP7, VP2, and VP3 genes. Remarkable lineage shift was observed for NSP1 gene; lineage A1-2 emerged in 2007 and became dominant in 2008–2009 epidemic season, while lineage A1-1 persisted throughout the study period.

**Conclusion:**

Chinese G3P[8] rotavirus strains have evolved since 2000 by intra-genogroup reassortment with co-circulating strains, accumulating more reassorted genes over the years. This is the first large-scale whole genome-based study to assess the long-term evolution of common human rotaviruses (G3P[8]) in an Asian country.

## Introduction

Group A rotavirus (RVA) is the leading etiological agent responsible for severe diarrhea in infants and young children worldwide, causing approximately 453,000 deaths each year [1]. Rotavirus, a genus of the family *Reoviridae*, has 11 segments of double-stranded RNA as its genome which are enclosed in a triple-layered capsid. These segments encode six structural proteins (VP1-VP4, VP6 and VP7) and six nonstructural proteins (NSP1-NSP6) [2]. Two outer capsid proteins, VP7 and VP4, contain neutralizing epitopes which define serotypes of rotavirus. Based on diversity of the VP7 and VP4 genes, G and P genotypes have been defined for RVA, respectively, and at least 27 G types and 37 P types have been discriminated [3], [4]. In human rotaviruses, G1, G2, G3, G4, G9, and G12 combined with P[4], P[6], and P[8] are frequently detected throughout the world, with G1P[8] being the most prevalent in humans [5], [6]. In terms of the whole genome-based genotyping system, most of human rotaviruses are classified into at least two major genogroups, i.e., Wa genogroup and DS-1 genogroup, with genotype constellations G1(or 3, 4)-P[8]-I1-R1-C1-M1-A1-N1-T1-E1-H1 and G2-P[4]-I2-R2-C2-M2-A2-N2-T2-E2-H2, respectively.

G3P[8], one of the common types in human rotaviruses, accounted for about 3.3–5.4% of all the strains from 1981 through 2004 globally, and had been described as the fourth dominant type following G1P[8], G2P[4], and G4P[8] [5], [6]. However, the proportion of G3P[8] among human RVA increased to 18.9% in Asia, in 2000–2009 [7], and G3P[8] became predominant or dominant genotype in eastern and south-east Asia from 2000 through 2011 [7]–[13]. These G3 strains were referred to as “the new variant G3” rotaviruses, represented by strain RVA/Human-wt/JPN/5091/2003-2004/G3P[X]. VP7 genes of the new variant G3 strains shared nucleotide sequence identities of ≤98% with those of conventional G3 rotaviruses. This was attributed to accumulation of mutations in the VP7 genes of these new variant RVAs, some of which resulted in amino acid changes [14], [15]. In China, G3P[8] has been reported as a dominant strain since the late of the 1990's in some provinces [8], [9], and became the most common genotype all over the country from 2000 through 2010 [8]–[13]. In Wuhan, a city located in central China, G3P[8] has been a predominant genotype from December 2000 through 2009–2010 epidemic seasons, then decreased to 10.2% during the 2011–2012 epidemic year [16]–[18]. It is suggested that the new variant G3 rotavirus emerged in the mainland of China in 1997 or earlier, thereafter spread in China and the areas around it in the following decade (Table S1). The G3P[8] rotaviruses with VP7 gene genetically close to the new variant G3 strain were detected also in Ireland, Spain, Canada, South Africa, America, Argentina, Germany, Italy, Belgium, Nicaragua from 2004 through 2010 [16]–[46]. These findings indicated that the new variant G3P[8] rotavirus might have emerged in Asia and rapidly spread worldwide.

To obtain conclusive data on the overall genetic makeup and evolutionary patterns of common RVAs, whole genomic analysis of rotavirus strains detected over a period of many years is essential [47]. Because the G3 RVA has been prevailing in China for more than 10 years, whole genome-based phylogenetic analysis may reveal genetic evolution of this common RVA and genetic mechanisms of their successful spread. However, for the new variant G3P[8] RVA strains, only the VP7 genes and their deduced amino acid sequences have been exclusively analyzed so far [14], [15], [21], [26], [30]. In China, whole genomic analysis of human RVA was performed for only three G1P[8] strains [48], a G2P[4] strain [49], two G3P[9] strains [50], and five G4P[6] strains [51], [52]. On the other hand, whole genome of G3P[8] RVA was analyzed only for those detected in the US in 1974–1980,1991 and 2006–2008 [19], [37]. Therefore, the present study in China is the first long-term large scale study on G3P[8] rotaviruses outside the US.

Conventionally, rotavirus genome has been detected and studied by migration pattern of the 11 segments in polyacrylamide gel electrophoresis (PAGE), i.e., RNA pattern or electropherotype. The polymorphism of the electropherotypes reflects the diversity of individual gene segments, and can be caused by mutations, rearrangement and reassortment of gene segments [2]. In previous reports, recent G3P[8] human RVA strains in Wuhan, China and Haiphong, Vietnam were discriminated into several electropherotypes [17], [33], suggesting the presence of heterogeneous viruses among the new variant G3P[8] rotaviruses.

To describe genetic diversity and evolution of the whole genome of G3P[8] human rotaviruses and to explore the possible origin of these RVA strains, we determined the whole genome sequences of 33 G3P[8] rotavirus strains with identical or different electropherotypes detected in Wuhan from 2000 through 2013. In this study, phylogenetic analysis was performed together with G3P[8] strains and the other common Wa genogroup strains with G1P[8], G9[8], G4P[8] genotypes worldwide, to know relatedness to these common RVA strains. Because of predominance of the G3P[8] rotavirus in China in the last decade, large-scale whole genome-based study of these rotavirus strains provides significant information on evolution of RVAs, which is relevant to vaccine practice.

## Materials and Methods

### Specimens and detection of rotavirus

Stool specimens were collected in Wuhan from December 2000 through May 2013 as described in previous studies [16]–[18]. The presence of rotaviruses in stool specimens was determined by detection of 11 RNA segments of rotavirus by PAGE as described previously [53]. Viral dsRNA was extracted from 400 μl of 10% stool suspension with sodium dodecyl sulfate (SDS) and phenol, and precipitated with ethanol. RNA segments of rotavirus were separated by PAGE and stained with silver nitrate and electropherotypes were discriminated as described previously [53].

### Genotyping of rotavirus

VP7 and VP4 genotyping of the RVA strains were performed by multiplex semi-nested PCR assays using several sets of G- and P- genotype primers as described previously [54]–[57]. The validity of the PCR-based genotyping data was confirmed by sequencing the VP7 and VP4 genes of several strains representing the different genotypes, such as G1, G2, G3, G4, G9, P[4], P[6], P[8] and P[9].

### Nucleotide sequencing and phylogenetic analysis

Nearly full-length nucleotide sequences (excluding the 5′-end and 3′-end primer sequences) of gene segments encoding VP7-VP4-VP6-VP1-VP2-VP3-NSP1-NSP2-NSP3-NSP4-NSP5/6 were determined directly with RT-PCR products. Viral RNA was extracted from stool samples or the tissue culture fluid using the QIAamp Viral RNA Mini Kit (Qiagen GmbH, Germany). Primers used for the amplification of different RVA genes are shown in Table S2. RT-PCRs were performed using the QIAGEN One Step RT-PCR Kit (Qiagen GmbH, Germany). Nucleotide sequences were determined using the BigDye Terminator v3.1 Cycle Sequencing kit (Applied Biosystems, CA, USA) on an automated DNA sequencer (ABI PRISM 3730). Phylogenetic trees of concatenated all the segments (concatenated ORF nucleotide sequences for each strain) and the individual segments were constructed by Maximum Likelihood method using MEGA (v5.01) software. The trees were statistically supported by bootstrapping with 1000 replicates, and phylogenetic distances were measured by Hasegawa-Kishino-Yano model. Phylogenetic analysis was also validated by other models, i.e., Kimura 2-parameter model, Jukes-Cantor model, and Tamura-Nei model. Multiple alignments of the determined sequences were performed using CLUSTAL W (http://clustalw.ddbj.nig.ac.jp/) and MAFFT (http://mafft.cbrc.jp/alignment/software/) program with default parameters.

### Accession numbers of nucleotide sequences

The nucleotide sequences of the 11 gene segments of the 33 G3P[8] RVA strains were deposited in the GenBank database under accession numbers KF371703-KF371942 and KF371662-KF371702, except for the VP7 genes of Y111, L210, R709, L478, E566 which were sequenced previously (Table 1). In addition to these G3P[8] strains, we also obtained the nearly full-length nucleotide sequences of the NSP1, NSP2, NSP3, and NSP4 genes of an archival G3 strain YO (RVA/Human-tc/JPN/YO/1976/G3P[8]) (GenBank accession numbers JX629046-JX629049, respectively).

**Table 1:** Profiles of Chinese G3P[8] RVA strains from 2000 to 2013.

No.	Strain	Date of collection (year-month)	sex	age (year, y/month, m) (child/adult)	PAGE profile of RNA segment 5	GenBank accession numbers	
						NSP1-NSP5/6,VP1-VP6	VP7
1	RVA/Human-wt/CHN/A16/2000/G3P[8]	2000-12	M	1.5 y	E-A1-1	KF371725–KF371734	KF371735
2	RVA/Human-wt/CHN/31/2002/G3P[8]	2002-9	F	child	E-A1-1	KF371703–KF371712	KF371713
3	RVA/Human-wt/CHN/723/2003/G3P[8]	2003-1	M	7 m	E-A1-1	KF371714–KF371723	KF371724
4	RVA/Human-wt/CHN/R107/2003/G3P[8]	2003-11	F	adult	E-A1-1	KF371954–KF371963	KF371964
5	RVA/Human-wt/CHN/R303/2004/G3P[8]	2004-1	M	4 y	E-A1-1	KF371965–KF371974	KF371975
6	RVA/Human-wt/CHN/Y106/2004/G3P[8]	2004-10	F	20 m	E-A1-1	KF371987–KF371996	KF371997
7	RVA/Human-tc/CHN/Y111/2004/G3P[8]	2004-10	F	9 m	E-A1-1	KF371693–KF371702	DQ873678
8	RVA/Human-tc/CHN/L148/2004/G3P[8]	2004-12	M	23 y	E-A1-1	KF371672–KF371681	KF371682
9	RVA/Human-wt/CHN/L210/2005/G3P[8]	2005-2	M	43 y	E-A1-1	KF371890–KF371899	DQ873671
10	RVA/Human-tc/CHN/R709/2005/G3P[8]	2005-8	F	3 m	E-A1-1	KF371683–KF371692	DQ873677
11	RVA/Human-wt/CHN/L478/2006/G3P[8]	2006-1	M	52 y	E-A1-1	KF371900–KF371909	EU708580
12	RVA/Human-wt/CHN/R1267/2006/G3P[8]	2006-9	M	3 y	E-A1-1	KF371921–KF371930	KF371931
13	RVA/Human-wt/CHN/E093/2007/G3P[8]	2007-3	F	10 m	E-A1-2	KF371736–KF371745	KF371746
14	RVA/Human-wt/CHN/E329/2007/G3P[8]	2007-6	F	1 y	E-A1-2	KF371747–KF371756	KF371757
15	RVA/Human-tc/CHN/E566/2007/G3P[8]	2007-9	M	1 y	E-A1-1	KF371662–KF371671	EU708585
16	RVA/Human-wt/CHN/E707/2007/G3P[8]	2007-10	F	1 y	E-A1-2	KF371758–KF371767	KF371768
17	RVA/Human-wt/CHN/E956/2008/G3P[8]	2008-2	F	8 m	E-A1-1	KF371769–KF371778	KF371779
18	RVA/Human-wt/CHN/E1367/2008/G3P[8]	2008-10	M	6 m	E-A1-2	KF371780–KF371789	KF371790
19	RVA/Human-wt/CHN/L1066/2009/G3P[8]	2009-2	F	64 y	E-A1-2	KF371910–KF371919	KF371920
20	RVA/Human-wt/CHN/E1857/2009/G3P[8]	2009-10	M	7 m	E-A1-2	KF371791–KF371800	KF371801
21	RVA/Human-wt/CHN/E1861/2009/G3P[8]	2009-10	F	9 m	E-A1-1	KF371802–KF371811	KF371812
22	RVA/Human-wt/CHN/E2000/2010/G3P[8]	2010-2	M	1 y	E-A1-1	KF371813–KF371822	KF371823
23	RVA/Human-wt/CHN/E2421/2010/G3P[8]	2010-12	F	1 y6 m	E-A1-1	KF371824–KF371833	KF371834
24	RVA/Human-wt/CHN/E2422/2010/G3P[8]	2010-12	M	6 m	E-A1-2	KF371835–KF371844	KF371845
25	RVA/Human-wt/CHN/E2432/2010/G3P[8]	2010-12	M	6 m	E-A1-2	KF371846–KF371855	KF371856
26	RVA/Human-wt/CHN/E2461/2011/G3P[8]	2011-1	M	1 y3 m	E-A1-1	KF371857–KF371866	KF371867
27	RVA/Human-wt/CHN/R1604/2011/G3P[8]	2011-1	F	8 m	E-A1-1	KF371976–KF371985	KF371986
28	RVA/Human-wt/CHN/Z1557/2011/G3P[8]	2011-10	F	62 y	E-A1-2	KF371998–KF372007	KF372008
29	RVA/Human-wt/CHN/E2835/2011/G3P[8]	2011-11	M	5 m	E-A1-2	KF371868–KF371877	KF371878
30	RVA/Human-wt/CHN/L1450/2012/G3P[8]	2012-1	F	51 y	E-A1-1	KF371943–KF371952	KF371953
31	RVA/Human-wt/CHN/Z1602/2012/G3P[8]	2012-1	M	57 y	E-A1-2	KF372009–KF372018	KF372019
32	RVA/Human-wt/CHN/E3239/2012/G3P[8]	2012-9	M	1 y	E-A1-1	KF371879–KF371888	KF371889
33	RVA/Human-wt/CHN/L1621/2013/G3P[8]	2013-3	F	57 y	E-A1-1	KF371932–KF371941	KF371942

## Results

### Prevalence and fluctuation of G types during the study period

**Figure 1:**
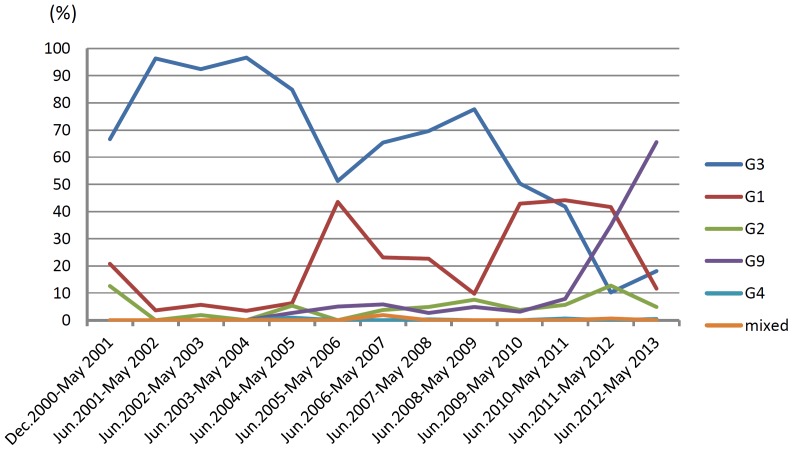
Fluctuation in proportion (%) of rotavirus G types in Wuhan, China, from 2000–2001 to 2012–2013 epidemic seasons.

From December 2000 through May 2013, the VP7- and VP4- genes of a total of 1644 RVA strains were genotyped. G3 was identified in 895 strains, among which 889 strains were typed as G3P[8], while remaining strains were found to have G3P[4], G3P[6] or G3P[9] genotypes. Genotypes of 89 G3, 63 G9, 48 G1, 10 G2, 4 G4, 105 P[8], 15 P[4], 5 P[6], and 2 P[9] strains were confirmed by sequencing of the VP7 and VP4 genes. G3 RVAs were predominant from 2000 through 2010. The proportion of G3 RVAs increased dramatically from 66.7% in 2000–2001 to 96.4% in 2001–2002, and was maintained over 84% until 2004–2005. However, it decreased to 51.3% in 2005–2006, then after slight increase in 2008–2009 (77.7%), decreased again gradually to 10.2% in 2011–2012 epidemic season (Fig. 1).

### Electropherotypes of Rotavirus strains and two distinct migration patterns of NSP1 gene

A total of 33 G3P[8] RVA strains were selected for the whole genomic sequencing analysis (Table 1). At least 2 strains in each epidemic year at the beginning and the end of the epidemic season (from September to the following February) were chosen for this analysis, except for 2001. During this study period, at least 14 electropherotypes of G3P[8] HRV were discriminated (data not shown). All the 889 G3P[8] strains were classified into two types on the basis of distinct migration patterns of RNA segment 5 (NSP1 gene) which are designated E-A1-1 and E-A1-2 with slower and faster migration, respectively (Fig. S1). The E-A1-1-NSP1 gene segment had been detected throughout the study period since 2000, while E-A1-2 segment was detected in 2007 and thereafter (Table 2). Strains with segment E-A1-2 as well as those with E-A1-1 were selected for whole genomic analysis after 2007.

**Table 2:** Frequency of G3P[8] RVA having RNA segment 5 (NSP1 gene) with different migration speed in PAGE.

period	segment 5*	
	E-A1-1	E-A1-2
Dec.2000–May 2001	16	0
Jun.2001–May 2002	27	0
Jun.2002–May 2003	49	0
Jun.2003–May 2004	28	0
Jun.2004–May 2005	95	0
Jun.2005–May 2006	39	0
Jun.2006–May 2007	60	7
Jun.2007–May 2008	158	60
Jun.2008–May 2009	51	90
Jun.2009–May 2010	66	15
Jun.2010–May 2011	48	25
Jun.2011–May 2012	12	5
Jun.2012–Apr. 2013	34	4
total	683	206

*E-A1-1 and E-A1-2 represent RNA segment 5 which migrated slower or faster in PAGE, respectively, as shown in Fig. S1.

### Genotype constellation and phylogenetic analysis of the G3P[8] RVA strains

**Figure 2:**
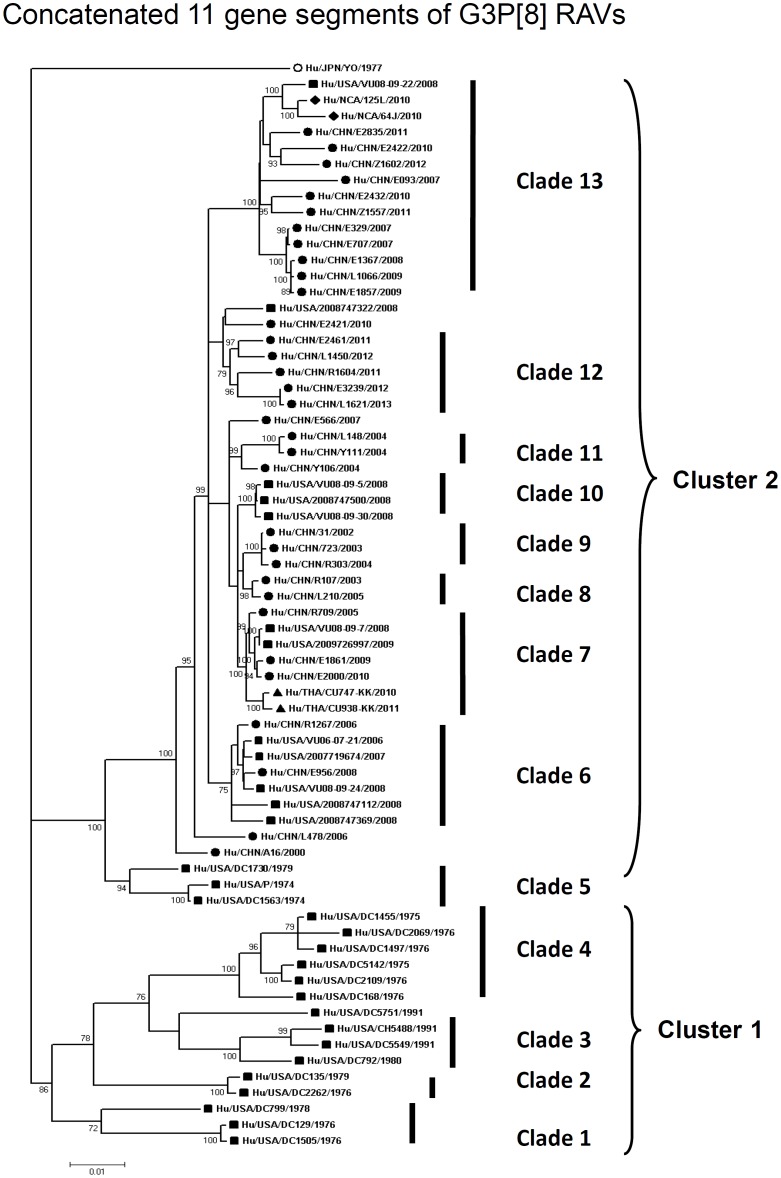
Phylogenetic dendrogram constructed from concatenated sequence of all the 11 RNA segments. Clusters and clades were assigned arbitrarily based on observation of clustering patterns, and shown on the right. In the phylogenetic tree of the concatenated sequences of G3P[8], G3P[8] strains analyzed in the present study are marked with circle, while other G3 P[8] strains with square, triangle and rhombus. Scale bars are shown below. Bootstrap values are indicated at nodes of branches.

**Figure 3:**
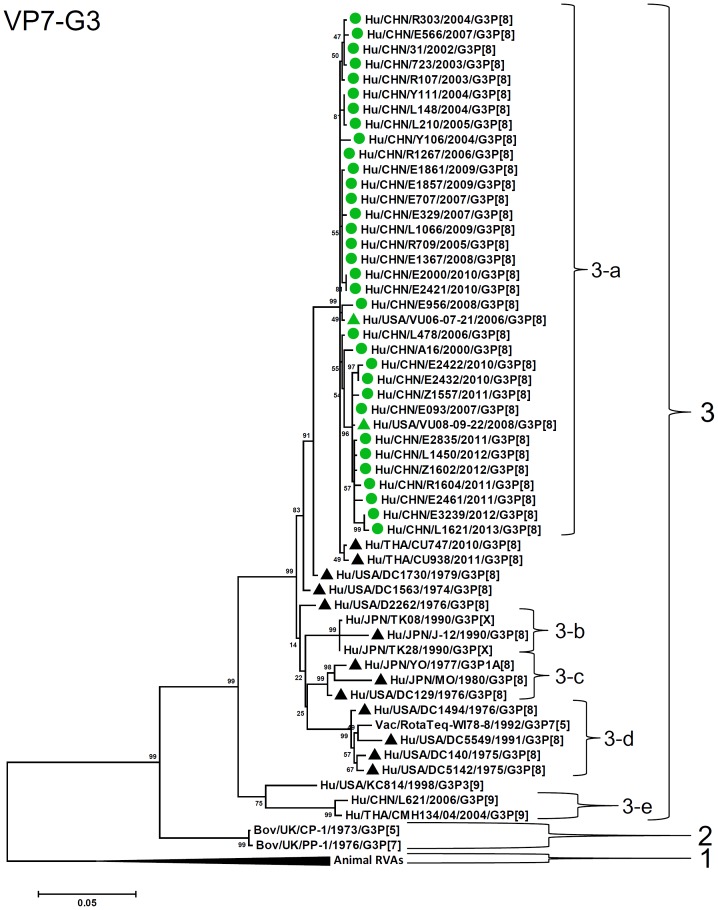
Phylogenetic dendrogram constructed from VP7 gene with genotypes G3. Lineages and sublineages within a genotype were assigned arbitrarily based on observation of clustering patterns, and shown on the right. G3P[8] strains analyzed in the present study are marked with circle, while G1P[8] strains in Wuhan, China [48], with square, and G3P[8] strains in the US reported previously [19], [37] and G3P[8] strain YO with triangle. Lineages (sublineages) of above strains are discriminated by colors of the marks, i.e., green (main lineage), orange, red, pink, blue, or gray. Black triangles indicate strains outside above lineages. Colors of lineages in each RNA segment correspond to those shown in Fig. 15. Scale bars are shown below. Bootstrap values are indicated at nodes of branches.

**Figure 4:**
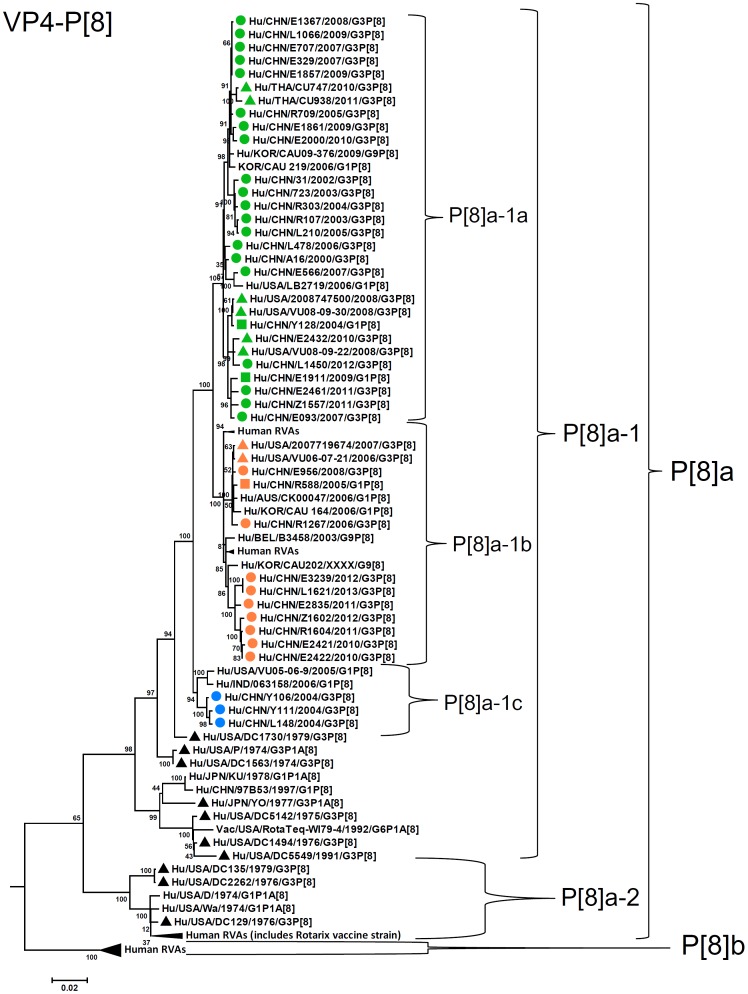
Phylogenetic dendrogram constructed from VP4 gene with genotypes P[8]. See legends of Fig. 3.

**Figure 5:**
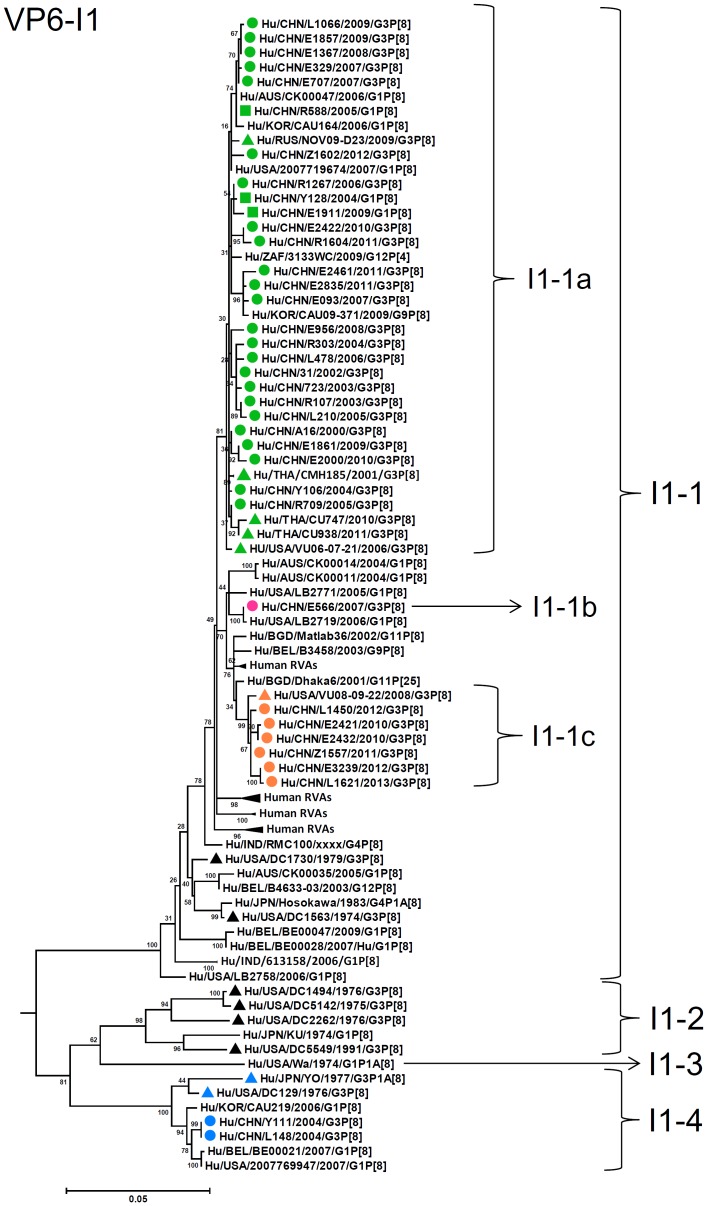
Phylogenetic dendrogram constructed from VP6 gene with genotypes I1. See legends of Fig. 3.

**Figure 6:**
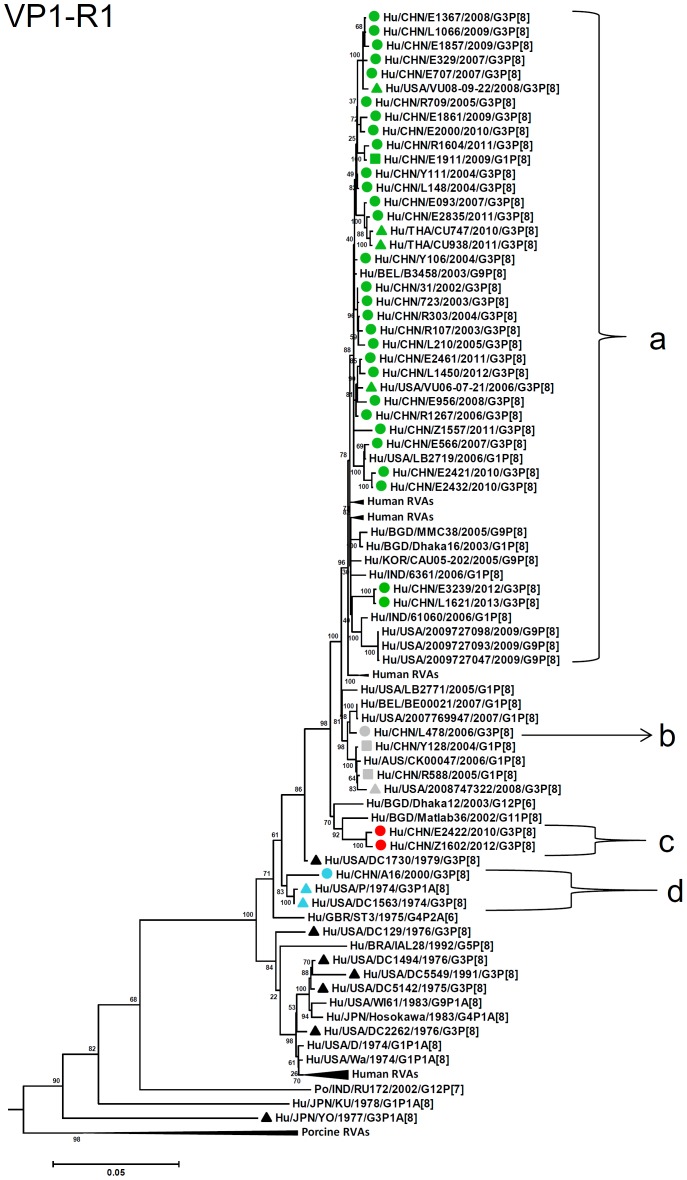
Phylogenetic dendrogram constructed from VP1 gene with genotypes R1. See legends of Fig. 3.

**Figure 7:**
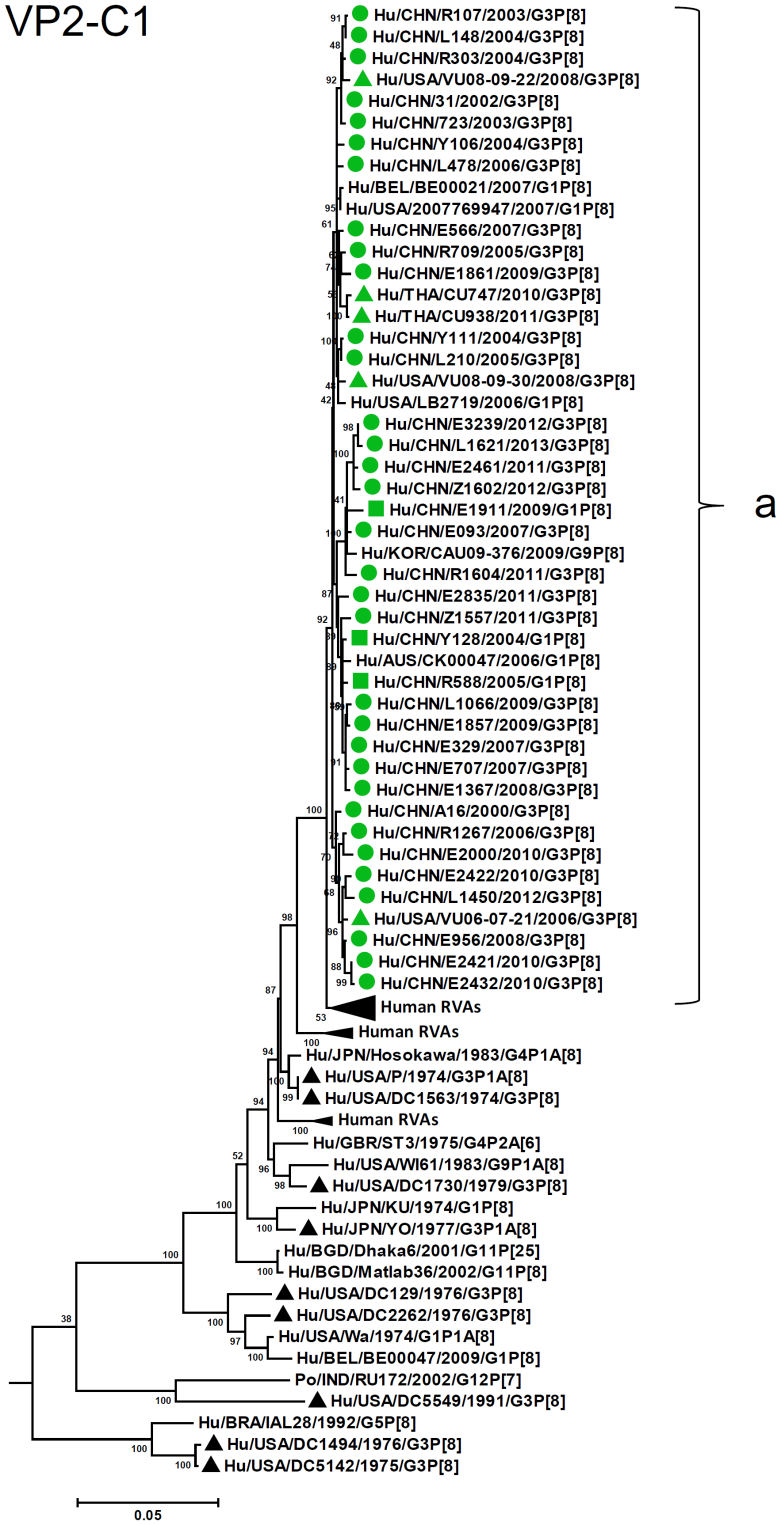
Phylogenetic dendrogram constructed from VP2 gene with genotypes C1. See legends of Fig. 3.

**Figure 8:**
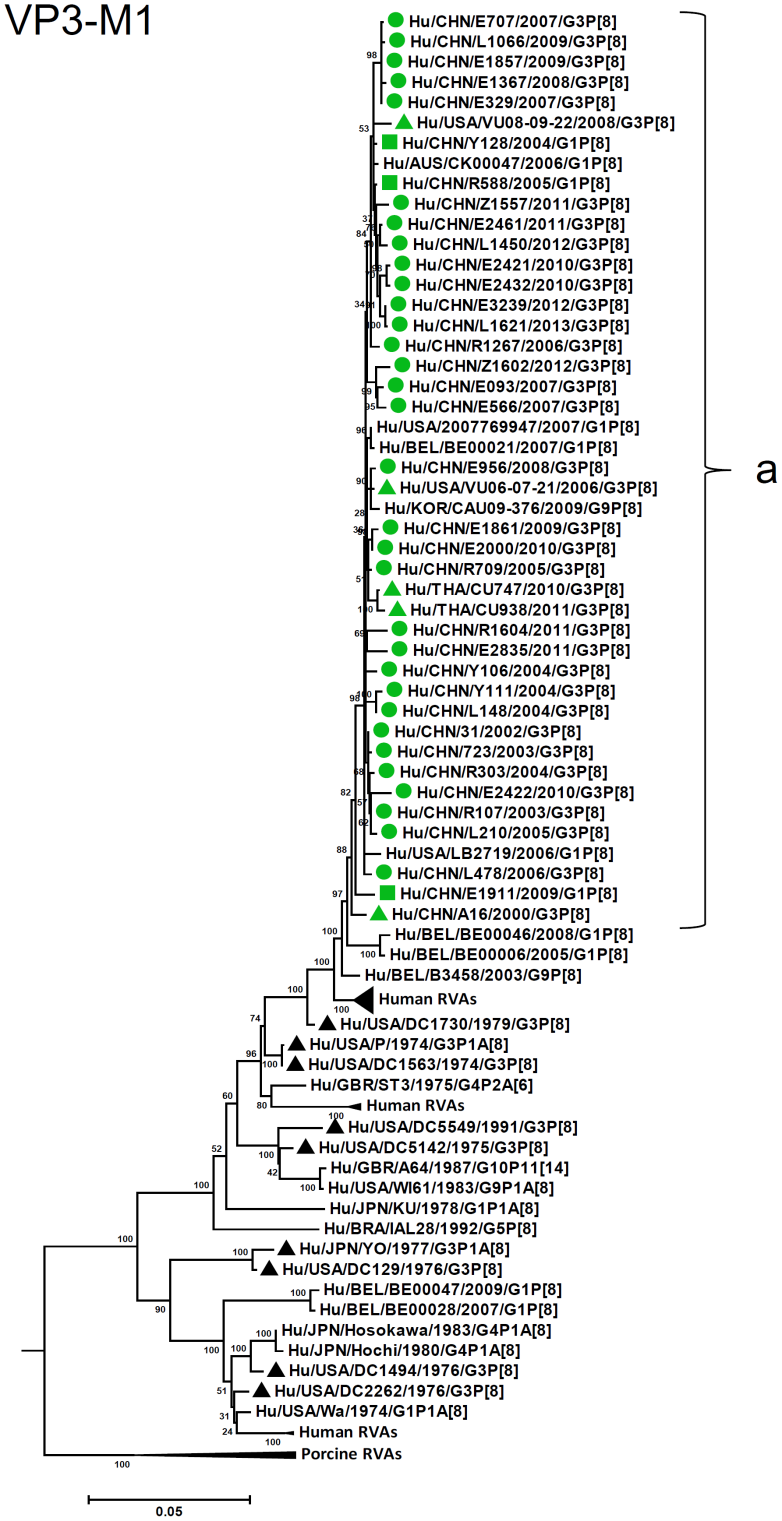
Phylogenetic dendrogram constructed from VP3 gene with genotypes M1. See legends of Fig. 3.

**Figure 9:**
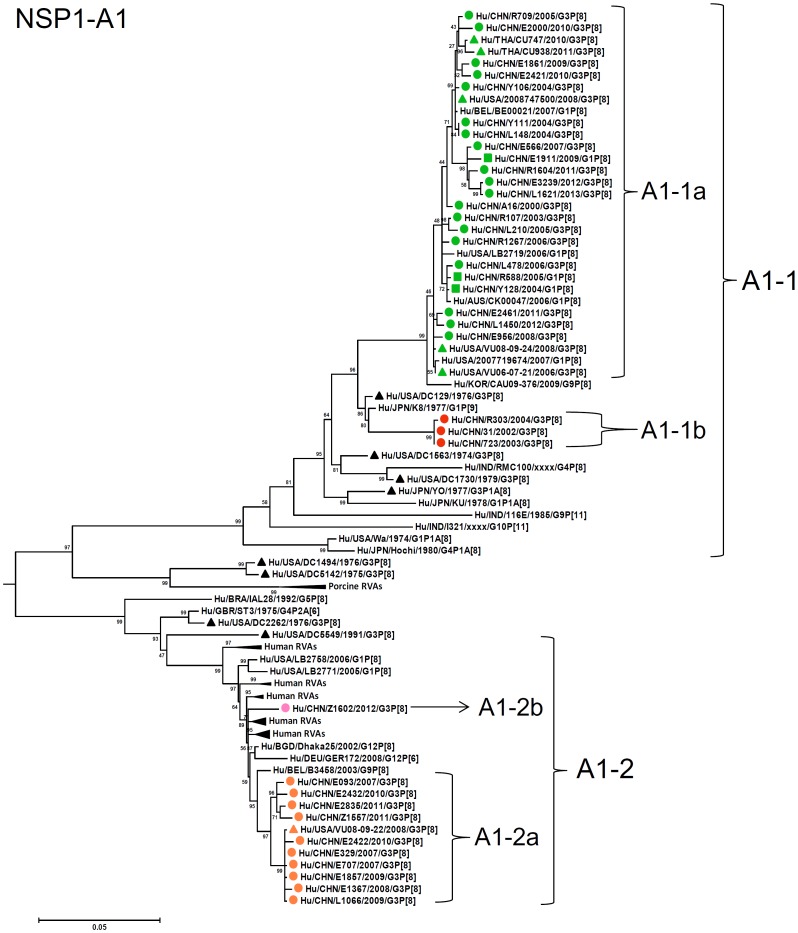
Phylogenetic dendrogram constructed from NSP1 gene with genotypes A1. See legends of Fig. 3.

**Figure 10:**
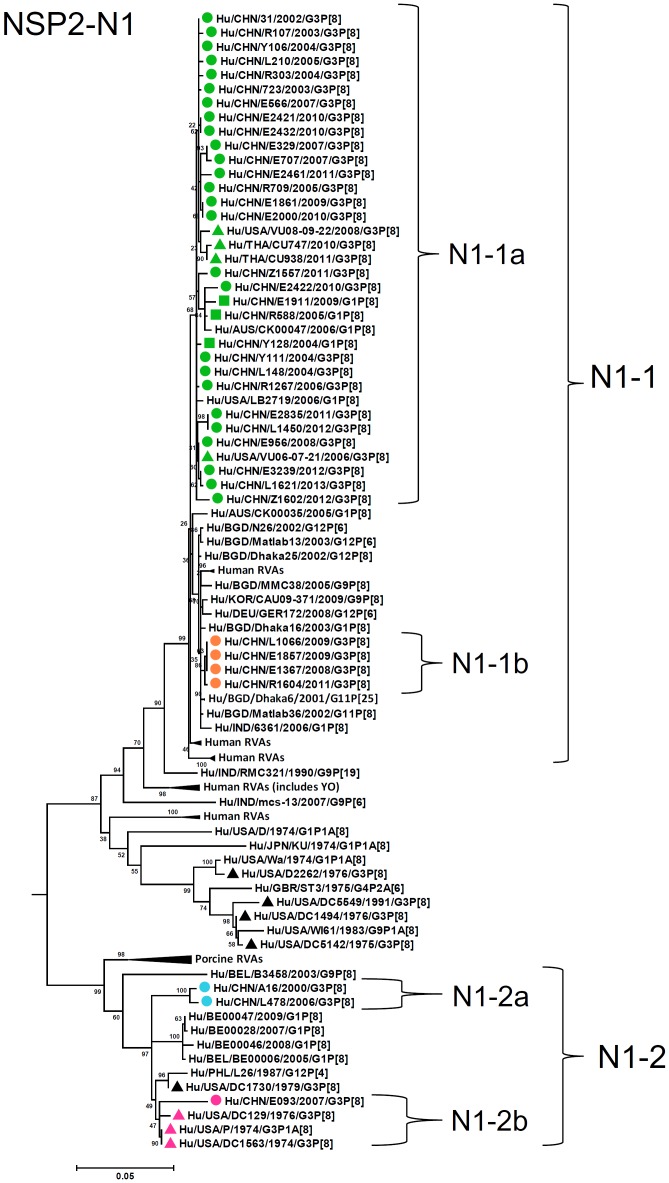
Phylogenetic dendrogram constructed from NSP2 gene with genotypes N1. See legends of Fig. 3.

**Figure 11:**
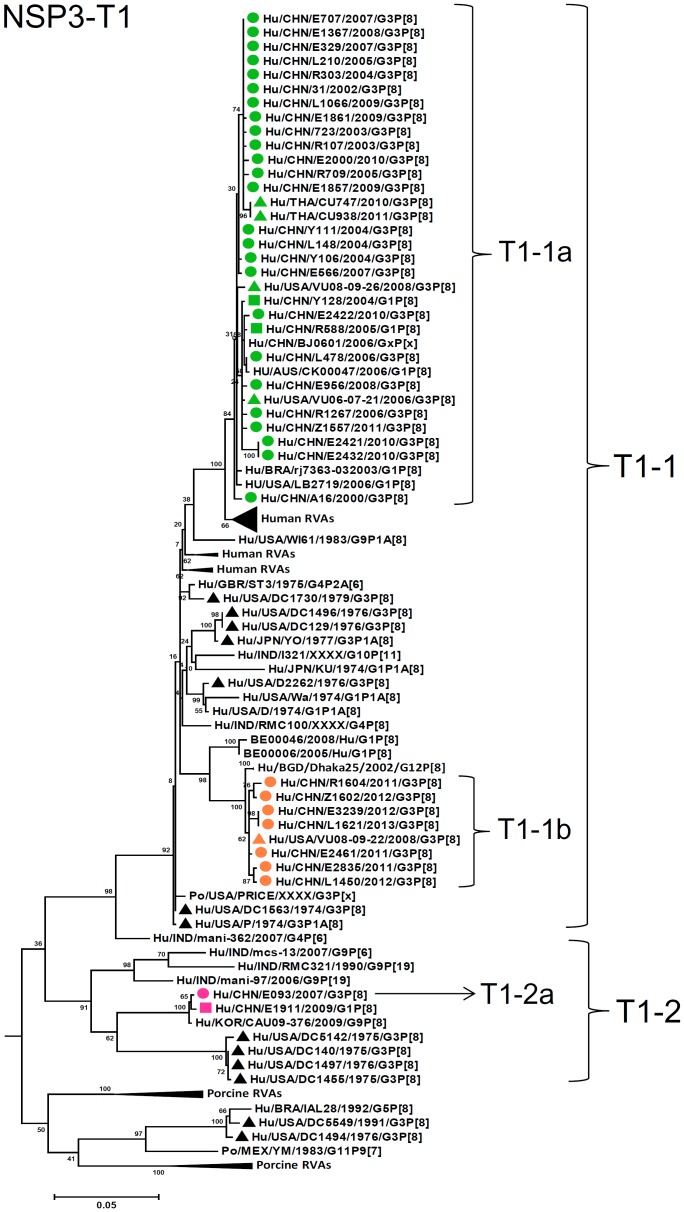
Phylogenetic dendrogram constructed from NSP3 gene with genotypes T1. See legends of Fig. 3.

**Figure 12:**
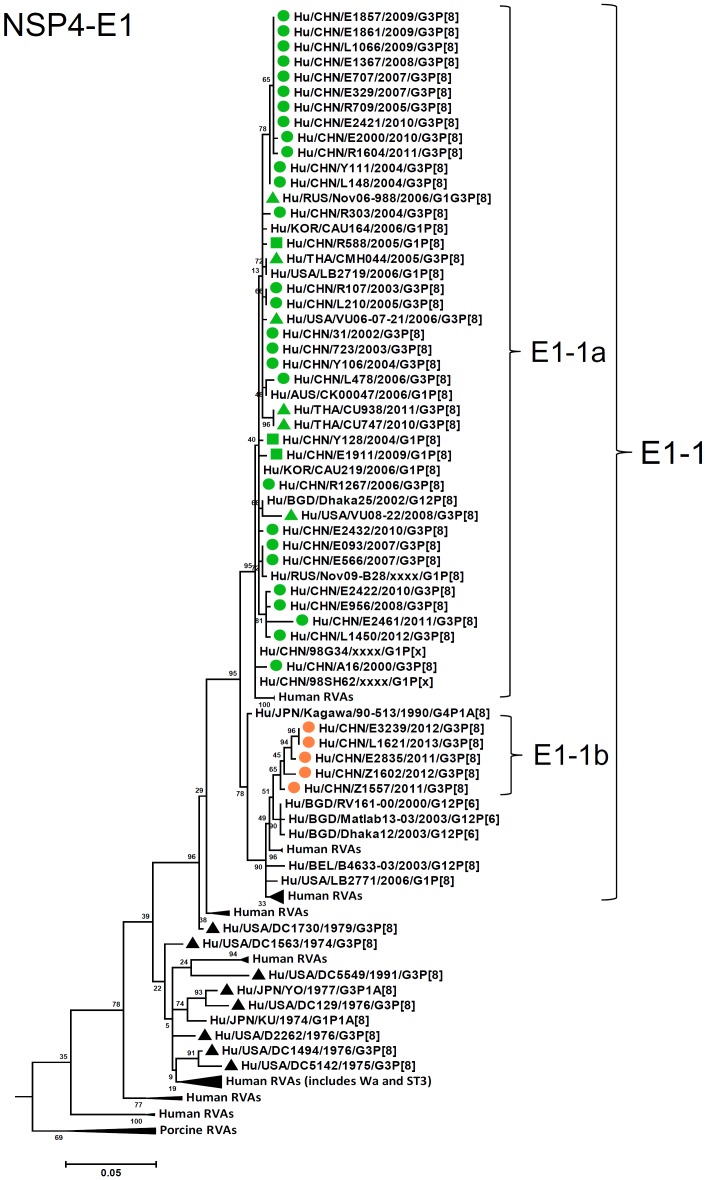
Phylogenetic dendrogram constructed from NSP4 gene with genotypes E1. See legends of Fig. 3.

**Figure 13:**
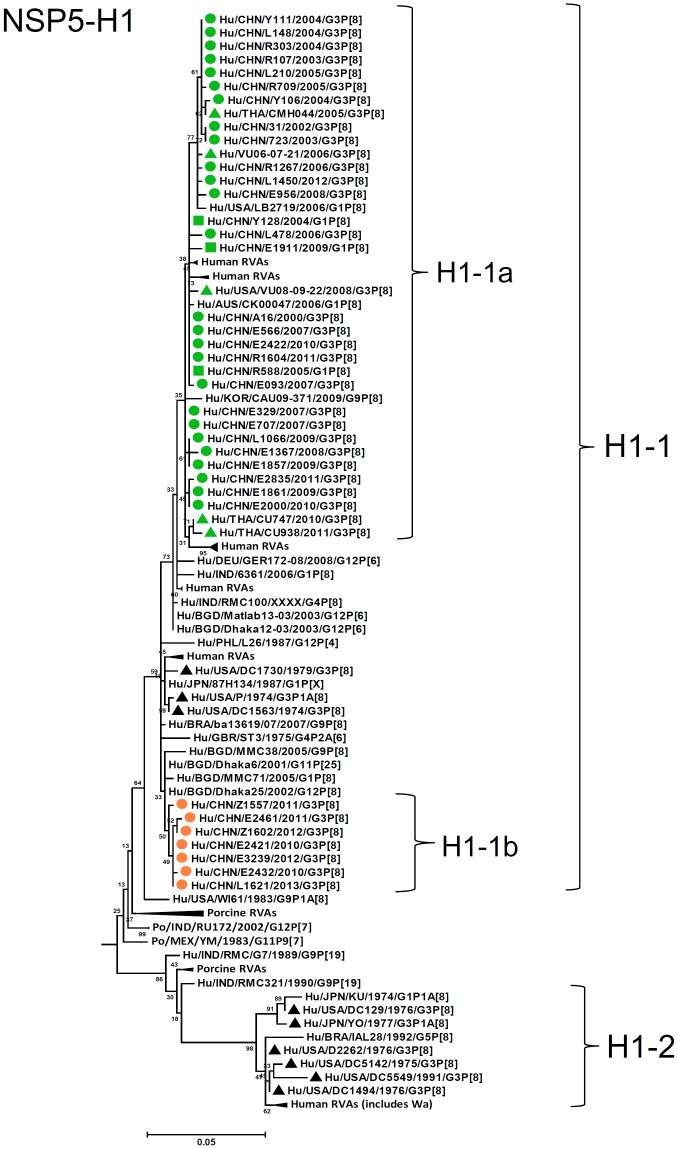
Phylogenetic dendrogram constructed from NSP1 gene with genotypes H1. See legends of Fig. 3.

**Figure 14:**
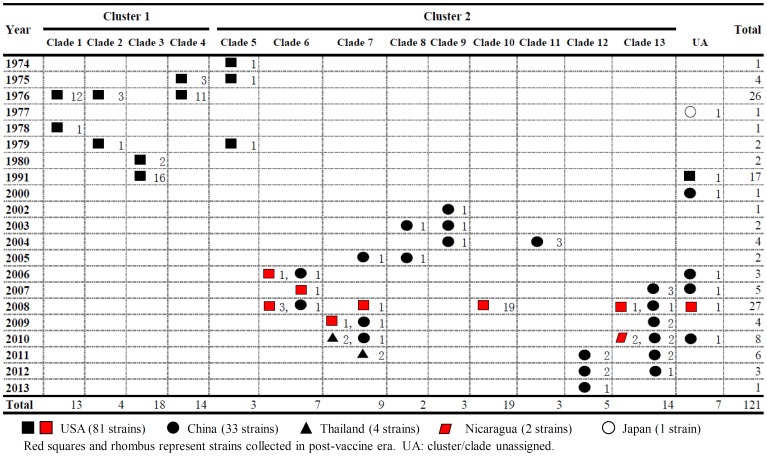
Temporal and spatial distributions of the clades of G3P[8] RVA genome of total 121 strains. Phylogenetic analysis of concatenated 11 gene segments was performed for 33 G3P[8] RVA strains in Wuhan, China, together with 88 strains in the world available in GenBank, to classify clade of each strain. Assigned clades shown on the top of this figure correspond to those in Fig. 2. Years of the detection of RVA strains are listed on the left. Symbols represent localities where the strains were collected, as shown in the bottom of the figure. The red symbols represent the strains detected in post-vaccine era. Numbers of the G3P[8] strains belonging to the same clades are shown together with the symbols.

By nucleotide sequence identities and phylogenetic analysis of the nearly full-length sequences of all the gene segments, the VP7-VP4-VP6-VP1-VP2-VP3-NSP1-NP2-NSP3- NSP4-NSP5/6 genes of all the 33 strains were assigned to G3-P[8]-I1-R1-C1-M1-A1-N1-T1-E1-H1 genotypes, respectively, indicating that all the G3P[8] RVA strains belonged to Wa genogroup. Among the 33 strains, the VP7, VP4, VP1-VP3, NSP4 and NSP5 genes were closely related with nucleotide sequence identities of 95–100%, while genetic diversity was noted for VP6 and NSP1-NSP3 genes (lowest identities of 83–89%). Phylogenetic trees were constructed for concatenated all the segments and individual RNA segments, and genetic relatedness was analyzed for the 33 G3P[8] strains together with other 88 G3P[8] strains and some strains with other genotypes belonging to Wa genogroup for which the whole genome sequences are available in GenBank database (Fig. 2–13). These 88 G3P[8] strains were collected in the US, Thailand, Nicaragua and Japan during various period(s) from 1974 through 2011. (Fig. 14) [19], [37].

In order to understand whole genomic relatedness of the Chinese G3P[8] strains and other G3 RVA strains for which the whole genome data are available, total 121 G3P[8] strains in the world were analyzed by the phylogenetic tree constructed using the concatenated ORF nucleotide sequences for each strain (Fig. 2 and Fig. 14). In the concatenated tree, the G3P[8] RVA strains were classified into 2 clusters. Cluster 1 contained archival G3P[8] strains in the US before 1991. All modern G3P[8] RVA in the world, including 33 Chinese G3P[8] strains detected from 2000 to 2013, were genetically grouped into cluster 2. In the cluster 2, 72 strains could be further classified as 9 clades (clade 5–13) with several exceptions. Chinese G3 RVA strains were classified into 7 clades (6–9, 11–13), among which recent Chinese G3 strains (2010–2013) were assigned into clades 7, 12, and 13, which clustered with a few G3P[8] strains in Thailand, the US and Nicaragua. Three old US strains in the 1970's clustered in clade 5 which is phylogenetically isolated from all other clades including Chinese G3 strains. Most of American modern G3P[8] strains were located in clade 10 that is close to the Chinese strains collected in 2002–2005. Some recent G3 strains in the US (2006–2008) clustered with Chinese G3 strains in clades 6, 7 and 13. Clade 13, including strains in China, the US and Nicaragua in 2007–2012, segregated consistently with the lineage A1-2 of NSP1 gene. A clade could persist for several years in different countries such as clades 6, 7 and 13, and different clades co-circulated in a same country.

G3-VP7 genes of the 33 strains were highly conserved (identities of 98–100%) and clustered into a single lineage (Lineage 3, sublineage 3a) with contemporary G3 strains in the world (Fig. 3). Most of other sublineages within lineage 3 (3b, 3c, and 3d) contained old G3 strains detected before 2000 and vaccine strain (WI78-7 of RotaTeq). P[8] (P[8]a)-VP4 genes showed 96–100% sequence identities and clustered into three sublineages of lineage 1 (1a, 1b, and 1c) (Fig. 4). Strains in sublineages P[8]a-1a and P[8]a-1b clustered with contemporary G3 RVA from other countries and G1 RVA strains detected in Wuhan, China, while sublineage P[8]a-1c comprises only three G3 strains detected in 2004.

VP6 genes of most of the G3 strains clustered into lineage I1-1, sublineages 1a, 1b, and 1c, with contemporary G3 strains from other countries and G1 strains in China, showing identities of 97–99% (Fig. 5). Two Chinese strains (2004) and the US strain DC799 (1978) were classified into lineage I1-4 with archival strain YO, showing 89–90% identities to strains in I1 sublineages 1a, 1b, and 1c.

R1-VP1 genes from the 33 G3 strains exhibited high sequence identities (95–99%) and were differentiated into four clusters (a–d), with cluster “a” containing majority of the strains (Fig. 6). VP2 and VP3 genes (C1, M1, respectively) were highly conserved (identities of 98–99% and 98–100%, respectively) and located in a single cluster in phylogenetic trees (Fig. 7, 8). However, in VP2 gene sequence, internal 6 nucleotide-duplication was detected at two sites between nucleotide no. 89 and 90 (one strain in 2005), and no. 134 and 135 (four strains detected after 2011), associated with insertion of two amino acids, KE and KN, respectively (Fig. S2, S3).

A1-NSP1 genes were discriminated into two lineages, A1-1 (sublineages 1a and 1b) and A1-2 (sublineages 2a and 2b), which corresponded to E-A1-1- and E-A1-2-NSP1 genes defined by PAGE, respectively (Fig. 9). Sublineages 1a and 1b contained the Chinese G3 strains detected from 2000 to 2013, and contemporary G3 strains in the US, Thailand, and G1 strains in China, while all the Chinese G3 strains in lineage A1-2 were detected after 2007 and clustered with a G9P[8] strain in Belgium, two G3P[8] strains in Nicaragua, some archival and contemporary G3P[8] strains in the US [19], [37]. Although high sequence identity was observed among G3 strains within lineage A1-1 (93–99%) or A1-2 (97–100%), identities between lineages A1 and A2 were lower (83–85%).

N1-NSP2 genes were differentiated into two lineages (N1-1 and N1-2) with high identities among each lineage (98–100% and 94–99%, respectively) and lower identities between these lineages (89–91%) (Fig. 10). Lineage N1-2 contained three strains in 2000, 2006, and 2007. Most of T1-NSP3 genes were classified into a single lineage T1-1, either of two sublineages (T1-1a and T1-1b), exhibiting 94–100% identity (Fig. 11). Only one strain (E093) had an NSP3 gene assigned to lineage T1-2 (sublineage T1-2a) clustered with a G1P[8] strain E1911 detected in Wuhan, China, and showed 89–90% identities to G3 stains in T1-1 lineage.

E1-NSP4 genes of all the strains were grouped into a single lineage (E1-1), in either of the two sublineages (1a and 1b), showing high sequence identities (96–100%) (Fig. 12). Sublineage E1-1b comprised five latest G3 strains from 2011. Similarly, H1-NSP5 genes were highly conserved (identities of 97–100%) and grouped into two sublineages (1a and 1b) in a single lineage H1-1 (Fig. 13). H1-1b was latest lineage containing Chinese strains from 2010 until 2013.

**Figure 15:**
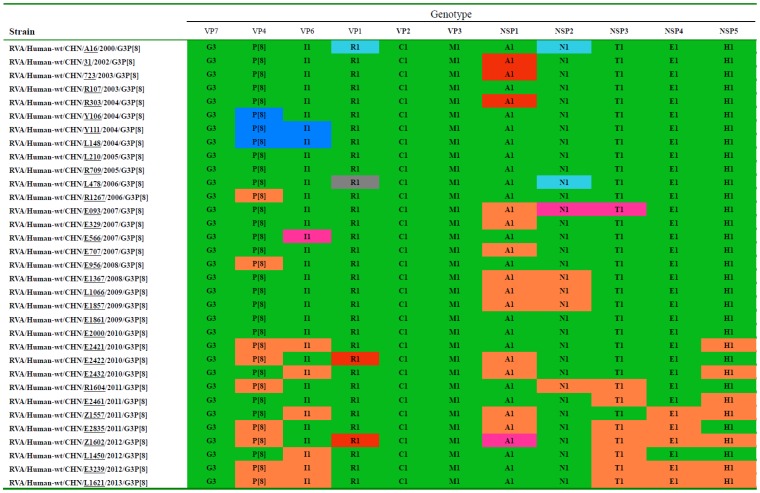
Genotype nature of the eleven gene segments of G3P[8] human rotavirus strains detected in Wuhan, China, from 2000 to 2013. Various colors have been used to highlight the different phylogenetic lineages or sublineages (assigned arbitrarily) observed within a genotype which correspond to those shown in Fig. 3–13. The common name of a strain is underlined.

Lineages in individual RNA segments of the 33 G3P[8] Chinese strains were summarized in Fig. 15 according to the year of detection. VP7, VP2, and VP3 genes belonged to a single lineage throughout the study period. Although almost no change of lineage was observed for VP1, NSP3, NSP4, and NSP5 genes until 2009, new lineages in these gene segments occurred in 2010 or 2011 and persisted thereafter. Late appearing sublineages in VP4 and VP6 genes (P[8]a-1b and I1-1c, respectively) were commonly found in strains detected after 2010. Since 2007, NSP1-A1-2 lineage has been detected together with conventional lineage NSP1-A1-1. The NSP1-A1-2 lineage was commonly associated with occurrence of new lineages in VP4, VP6, VP1, NSP2-NSP5 genes, although no fixed combination was detected. Co-occurrence of minor lineages in different RNA segments was found in older strains, i.e., A16 and L478 (VP1 and NSP2 genes), and Y111 and L148 (VP4 and VP6 genes).

### Amino acid difference among lineages of G3 strains

Amino acid difference in the deduced sequences of 11 viral proteins among different lineages of the 33 G3P[8] strains were investigated (Table S3), except for VP7, VP2, and VP3 with a single lineage. NSP4 and NSP5, and VP6 showed amino acid difference in only 3–7 positions among different lineages. The most diversity was detected in NSP1 at 110 amino acid positions. The divergent positions in NSP1, as well as those in NSP2 (21 sites) and NSP3 (33 sites), were generally scattered from N to C terminus of these proteins. However, this was not the case in VP1 and VP4, and amino acid diversity was not detected in specific regions in these proteins, e.g., amino acid no. 658–820 in VP1, and no. 339–544 in VP4.

### Comparison of VP7 and VP4 amino acid sequences to those of vaccine strains

Amino acid residues in the surface of VP7 and VP4 responsible for neutralization have been identified by analysis of neutralization escape mutants [45], [58], [59]. Alignment of the amino acid residues defining the VP7 neutralization domains of G3P[8] RVA strains with G3 component (WI78-9) of pentavalent rotavirus vaccine (RotaTeq) revealed that all the Chinese G3 strains had different amino acids at three among the six positions (212, 238, and 242) in neutralization domain 7-1b (Fig. S4). In VP8*, amino acids in two sites in neutralization domain 8-1, and a site in domain 8-3 of most of the G3 strains were different from those of two vaccine strains (Rotarix and RotaTeq) (Fig. S5). At some more sites in domains 8-1 and 8-3, amino acid residues of all the G3 strains were identical to RotaTeq, but different from Rotarix. Although VP5* sequences of G3 strains were more close to those of vaccine strains, at the two positions in domain 5-1, amino acids distinct from two vaccine strains were shared by all the 33 G3 strains (Fig. S6).

## Discussion

Rotaviruses are distributed globally as a major etiologic agent of infantile gastroenteritis, causing high mortality attributable to diarrhea in many African and Asian countries [1]. In terms of a global estimation, China ranked the fifth major country with rotavirus-related death [60], although estimated mortality reduced in the latest report on disease burden of rotavirus infection in Asia [7]. For prevention of severe symptoms due to rotavirus infection in children, two live vaccines, Rotarix and RotaTeq, are available and recommended for use in all countries by WHO. However, in China, these vaccines have not yet been available, although a phase III trial was conducted recently [61], and a lamb rotavirus vaccine LLR (G10P[12]) is practically used [62]. Antigenic and genetic characterizations of dominant wild rotavirus strains are essential for prediction of potential efficacy of any rotavirus vaccine, and would be also useful for development or improvement of vaccines. In the present study, we first disclosed whole genetic sequences and their characteristics of the predominant G3 rotaviruses in China.

As observed in the concatenated tree (Fig. 2), all the Chinese G3P[8] rotaviruses detected from 2000 to 2013 were genetically grouped into a single cluster (cluster 2), which was distinct from another cluster comprising G3 strains in the US before 1991 (cluster 1). Within the cluster 2, three old US strains in the 1970's clustered in clade 5 which is phylogenetically isolated from all other clades including Chinese G3 strains. Some of the recent US G3 strains in 2006–2008 (e.g., VU-08-09-30) were genetically close to Chinese strains in 2006–2012, and belonged to the new variant G3 rotavirus (Table S1). These findings suggest that genome of G3 strains might have evolved as a whole chronologically, and modern G3 rotaviruses in China, Thailand, the US and Nicaraguan are genetically related to some old G3 rotavirus in the US (clade 5 in cluster2). When combined with the phylogenetic trees (Fig. 3–13) of each segment, it is possible to consider that the new variant G3 rotavirus might be derived from one of the old G3 strains, such as those classified into clade 5, occurred in Asia in the late 1990's, and evolved through genetic drift and intra-genotype reassorment of NSP2 gene, spreading globally thereafter.

In the present study, evolutionary pattern of G3 RVA strains in China between 2000 and 2013 was elucidated. While main lineages of individual gene segments persisted during the study period (Fig. 15, lineages with green), different lineages appeared occasionally in RNA segments encoding VP1, VP4, VP6, and NSP1-NSP5, exhibiting various allele constellations. No definite pattern was found for combination of RNA segments with non-major lineages, except for a few strains, e.g., strains Y111 and L148 having VP4 and VP6 genes with the same, minor lineages (P[8]a-1c, I1-4, respectively) (Fig. 15). Although the Chinese G3 RVA strains before 2009 had all, or at least 8 RNA segments belonging to main lineages, all the analyzed strains after 2010 contained 2–6 RNA segments with non-major lineages. Some of the RNA segments with minor lineages were revealed to be genetically related to non-G3 RVA strains. For example, minor lineage P[8]a-1c of VP4 gene and I1-4 of VP6 gene found in strains Y111, L148 and DC799 clustered with contemporary G1P[8] strains, suggesting that these genes were introduced from other RVA such as G1P[8] strain via reassortment. Hence, the Chinese G3P[8] RVA strains are considered to have evolved since 2000 by intragenogroup reassortment with co-circulating strains, which occurred randomly in most RNA segments, accumulating more reassorted RNA segments over the years. However, only a single cluster was found for VP7, VP2, VP3 genes throughout the study period, indicating that no reassortment event has occurred for these genes. Based on these observations, it may be speculated that these viral structural genes were more stable compared to the other RVA genes. Only a mutation in VP2 gene, i.e., duplication of 6-nucleotide sequence was detected in some strains in 2005 and after 2011. This mutation was not related to any specific allelic constellation. Despite with a similarity to rearrangement which causes partial duplication of gene segments [47], this mutation appears to be a novel evolutionary event distinct from rearrangement because only a short nucleotide sequence is duplicated within an amino acid coding region of a structural protein gene, in RVA strains detected in common diarrheal specimens.

Evolutionary pattern of the G3 RVA in the US from 1974 to 1991, and 2007–2009 reported by McDonald et al. [19], [37] appears to be different from the Chinese G3 strains described above. G3 RVAs in the US, belonging to Wa-like genotype constellation, were discriminated into four major clades with distinct lineages in the whole RNA segments. Three clades persisted from 1974 to 1979, and a single clade was detected in 1991, as well as in 2007–2009, and reassortant among different clades was rarely identified. In contrast, G1P[8] RVA from 2006–2009 comprised with strains with various allele constellations, showing occurrence of intragenogroup reassortment among the co-circulating strains. In these studies, G1P[8] was described as the major genotype in the study period from 1974–1976, 1977–1989, and 2005–2008, while G3 was dominant occasionally, in 1976, 1991, 2008–2009. Thus, heterogeneity via reassortment was observed mostly among dominant genotype, i.e., G1P[8] strain in the US. Similarly, in China, RVA with dominant type G3P[8] was revealed to be heterogeneous in the present study, although RVA with other genotypes have not yet been well characterized. It is suggested that the rotavirus strains with dominant genotype may transmit among population more frequently than other genotypes, and have more chance to cause mixed infection with other rotaviruses belonging to different clades within the same genotype, yielding reassortants with various allelic constellation. Persistence of specific allele constellation in nature may be related to various factors, e.g., fitness of the whole gene segments, replication efficacy of the resultant viruses, and immune response to viral proteins, which remain to be elucidated.

It was of note that distinct NSP1 lineage (A1-2) emerged among G3 rotaviruses in 2007, while conventional lineage (A1-1) which is found in common RVA worldwide persisted throughout the study period. Phylogenetically, A1-2 NSP1 gene of the Chinese G3P[8] RV strains clustered with G3 strains in the US (1975–1991, 2008) and Nicaragua (2010), G1, G9, G12 RVA strains detected worldwide. Although origin of the A1-2 NSP1 gene is not evident, this gene is suggested to be derived from circulating human RVA strains with common genotypes (Fig. 9), and actually A1-1 and A1-2 are considered as two major lineages of A1-NSP1 gene among human rotaviruses. Although the A1-2 NSP1 gene (E-A1-2-segment 5 in PAGE) surpassed A1-1 in the 2008–2009 season, thereafter its frequency decreased gradually. NSP1, one of the RNA-binding proteins [2], is known to be highly divergent in its sequence among rotaviruses proteins, except for a conserved N-terminus cysteine-rich motif [63]. NSP1 is implicated in evasion of innate immune response by inhibiting induction of interferon (IFN) and NFκB activation, and delaying early cellular apoptosis [64]–[66]. Cellular type I IFN response is suppressed by function of NSP1 as an E3 ubiquitin ligase, through proteasome-dependent degradation of IFN regulatory factors (IRF)-3, -5, and -7 [64], [67]. Different suppression levels for type I IFN response as well as targeted molecules by NSP1 were observed depending on rotavirus strains having genetically distinct NSP1 genes [68], [69]. Although such a functional difference has not yet been elucidated by NSP1 genes belonging to different lineages within a single genotype, it is possible that the lineage switching in NSP1 gene over the years observed in the present study might be related to certain functional difference of NSP1 with different lineages, associated with immune response to dominant lineage of NSP1.

It was noted that NSP3 gene of a single G3 strain E093 in 2007 (lineage T1-2) was close to that of G1P[8] strain E1911 detected in Wuhan, China, in 2009 [48]. As observed for the strain E1911, NSP3 genes of these strains clustered near those of porcine-like human G9 strains in India in the lineage T1-2. Thus, it is suggested that G3 strain E093 might have acquired its NSP3 gene via intragenotype reassortment, possibly from porcine or porcine-like human RVAs. The fact that such porcine-like NSP3 gene was detected in G1 and G3 strains suggests persistence and potential spread of the gene segment of the zoonotic origin among common genotypes of human rotaviruses. To determine its significance, further surveillance of rotavirus may be required for the NSP3 gene of local human and porcine rotaviruses.

Compared with the two vaccine strains, some mismatches were found in deduced VP7 and VP4 (VP8* and VP5*) amino acid sequences of the G3 rotavirus strains in China, as reported for wild human RVA strains detected in the 2000's [45], [48]. In contrast to VP7 of G1P[8] RVA in Wuhan, China, or in Belgium, exhibiting mismatched amino acids located mostly in antigenic epitope 7-1a compared with vaccine strains, G3 strains in the present study showed amino acid divergence mostly in epitope 7-1b compared with G3 component of RotaTeq, which was similar finding to G3 strains in Belgium [45]. In VP4 sequence of G3 rotaviruses in the present study, most of mismatched amino acids and their locations compared with two vaccine strains were similar to those found in G1 strains in China, and G1 and G3 strains in Belgium (positions 146, 190, 196, 113, 125, 131, 135, 384, 386). These findings suggest that the VP7 and VP4 of recent human RVA have been subjected to similar genetic evolution with regard to antigenic regions, from old rotavirus strains which include the components in the current vaccines. In China, phase III trial of Rotarix conducted recently showed substantial level of protection against severe rotavirus gastroenteritis in children less than two years of age [61]. Therefore, it is suggested that amino acid diversity in VP7 and VP4 observed in modern G3 rotaviruses might not affect efficacy of the rotavirus vaccine. Nevertheless, by global introduction and use of rotavirus vaccines, it is suggested that more amino acid substitutions may occur through escape from neutralizing antibody evoked to the vaccine strains, which is suggested to influence the efficacy of current rotavirus vaccine. Hence, monitoring of the VP4 and VP7 sequences of common wild strains will be significant to speculate on the potential effectiveness of vaccine strains. In addition to antibody response to VP4 and VP7, antibodies elicited against VP2, VP6, NSP2, and NSP4 are also considered to confer protection to host against rotavirus infection [70]; accordingly, genetic monitoring of these genes would be also important.

In conclusion, this is the first large-scale whole genome-based study to assess the long-term evolution of common human RVA (G3P[8]) in an Asian country. The genetic information in this study is expected to contribute as a baseline data to understand long-term evolution of rotavirus genome, and to formulate policies for the use of rotavirus vaccines.

## Supporting Information

Figure S1 Electropherotypes of representative rotavirus strains analyzed in this study having distinct migration patterns of NSP1 gene (RNA segment 5).
(PPT)

Figure S2 Alignment of partial nucleotide sequences of VP2 gene of the G3 rotavirus strains.
(DOC)

Figure S3 Alignment of partial VP2 amino acid sequences of the G3 rotavirus strains.
(DOC)

Figure S4 Alignment of the amino acid residues defining the neutralization domains (designated as 7-1a, 7-1b and 7-2) of VP7 between the G3 strain in RotaTeq and Chinese G3P[8] RVA strains detected in Wuhan from 2000 to 2013. Red indicates the residues different from those of RotaTeq.
(DOC)

Figure S5 Alignment of the amino acid residues corresponding to those defining the VP4 neutralization domains (designated as 8-1, 8-2, 8-3 and 8-4) in the VP8* subunit between the P[8] strains in Rotarix and RotaTeq and Chinese G3P[8] RVA strains detected in Wuhan from 2000 to 2013.
(DOC)

Figure S6 Alignment of the amino acid residues corresponding to those defining the VP4 neutralization domains designated as 5-1, 5-2, 5-3, 5-4 and 5-5 in the VP5* subunit between the P[8] strains in Rotarix and RotaTeq and Chinese G3P[8] RVA strains detected in Wuhan from 2000 to 2013.
(DOC)

Table S1 Identity of VP7 gene and deduced amino acid sequences of representative G3 strains detected worldwide to the new variant G3 strain “5091”.
(XLS)

Table S2 Primers used for amplification of different genes of G3P[8] RVA strains.
(DOC)

Table S3 Amino acid differences among the Chinese G3P[8] RVA strains belonging to different phylogenetic lineages.
(DOC)
