# Supplementary material for: Molecular Epidemiology and Genetic Evolution of the Whole Genome of G3P[8] Human Rotavirus in Wuhan, China, from 2000 through 2013
Source: PLoS One. 2014 Mar 27;9(3):e88850. doi: 10.1371/journal.pone.0088850 (PMC3967987; doi:10.1371/journal.pone.0088850)
Supplement: Figure S1 — Electropherotypes of representative rotavirus strains analyzed in this study having distinct migration patterns of NSP1 gene (RNA segment 5). (PPT) [file pone.0088850.s001.ppt]

## Slide 1
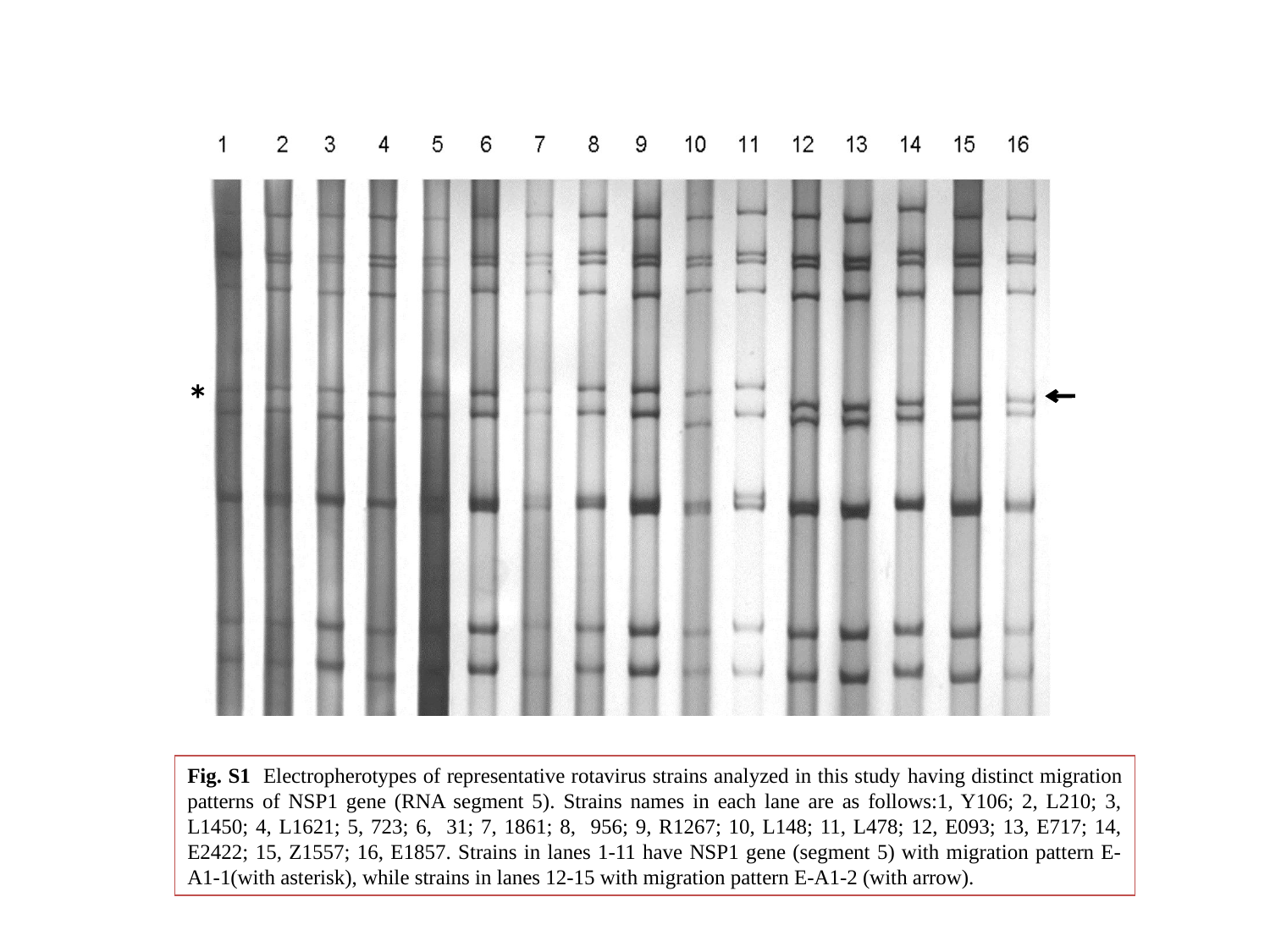

*
Fig. S1 Electropherotypes of representative rotavirus strains analyzed in this study having distinct migration patterns of NSP1 gene (RNA segment 5). Strains names in each lane are as follows:1, Y106; 2, L210; 3, L1450; 4, L1621; 5, 723; 6, 31; 7, 1861; 8, 956; 9, R1267; 10, L148; 11, L478; 12, E093; 13, E717; 14, E2422; 15, Z1557; 16, E1857. Strains in lanes 1-11 have NSP1 gene (segment 5) with migration pattern E-A1-1(with asterisk), while strains in lanes 12-15 with migration pattern E-A1-2 (with arrow).
