# Supplementary material for: Molecular Epidemiology and Genetic Evolution of the Whole Genome of G3P[8] Human Rotavirus in Wuhan, China, from 2000 through 2013
Source: PLoS One. 2014 Mar 27;9(3):e88850. doi: 10.1371/journal.pone.0088850 (PMC3967987; doi:10.1371/journal.pone.0088850)
Supplement: Figure S2 — Alignment of partial nucleotide sequences of VP2 gene of the G3 rotavirus strains. (DOC) [file pone.0088850.s002.doc]

**Fig.S2** Alignment of nucleotide sequences in partial VP2 gene of the G3 rotavirus strains. Nucleotides in green represent an inserted sequence which is a duplication shown in blue. Dash denotes gap, asterisk below alignment shows consensus nucleotide. Nucleotide numbers are shown above and right.

90 100

31VP22002 ACAAAATGAACGTCTGCAAGAAAAAGAAA------TTGAAAATAATACAGACGTAGCCAT 114

723VP22003 ACAAAATGAACGTCTGCAAGAAAAAGAAA------TTGAAAATAATACAGACGTAGCCAT 114

A16VP22000 ACAAAATGAACGTCTGCAAGAAAAAGAAA------TTGAAAATAATACAGACGTAATCAT 114

E093VP22007 ACAAAATGAACGTCTGCAAGAAAAAGAAA------TTGAAAATAATACAGACGTAACCAT 114

E329VP22007 ACAAAATGAACGTCTGCAAGAAAAAGAAA------TTGAAAATAATACAGACGTAACCAT 114

E566VP22007 ACAAAATGAACGTCTGCAAGAAAAAGAAA------TTGAAAATAATACAGACGTAACCAT 114

E707VP22007 ACAAAATGAACGTCTGCAAGAAAAAGAAA------TTGAAAATAATACAGACGTAACCAT 114

E956VP22008 ACAAAATGAACGTCTGCAAGAAAAAGAAA------TTGAAAATAATACAGACGTAACCAT 114

E1367VP22008 ACAAAATGAACGTCTGCAAGAAAAAGAAA------TTGAAAATAATACAGACGTAACCAT 114

E1857VP22009 ACAAAATGAACGTCTGCAAGAAAAAGAAA------TTGAAAATAATACAGACGTAACCAT 114

E1861VP22009 ACAAAATGAACGTCTGCAAGAAAAAGAAA------TTGAAAATAATACAGACGTAACCAT 114

E2000VP22010 ACAAAATGAACGTCTGCAAGAAAAAGAAA------TTGAAAATAATACAGACGTAACCAT 114

E2421VP22010 ACAAAATGAACGTCTGCAAGAAAAAGAAA------TTGAAAATAATACAGACGTAACCAT 114

E2422VP22010 ACAAAATGAACGTCTGCAAGAGAAAGAAA------TTGAAAATAATACAGACGTAACCAT 114

E2432VP22010 ACAAAATGAACGTCTGCAAGAAAAAGAAA------TTGAAAATAATACAGACGTAACCAT 114

E2461VP22011 ACAAAATGAACGTCTGCAAGAAAAAGAAA------TTGAAAATAATACAGACGTAACCAT 114

E2835VP22011 ACAAAATGAACGTCTGCAAGAAAAAGAAA------TTGAAATTAATACAGACGTAACCAT 114

E3239VP22012 ACAAAATGAACGTCTGCAAGAAAAAGAAA------TTGAAAATAATACAGACGTAACCAT 114

L148VP22004 ACAAAATGAACGTCTGCAAGAAAAAGAAA------TTGAAAATAATACAGACGTGGCCAT 114

L210VP22004 ACAAAATGAACGTCTGCAAGAAAAAGAAAAAGAAATTGAAAATAATACAGACGTAACTAT 120

L478VP22006 ACAAAATGAACGTCTGCAAGAAAAAGAAA------TTGAAAATAATACAGACGTAACCAT 114

L1066VP22009 ACAAAATGAACGTCTGCAAGAAAAAGAAA------TTGAAAATAATACAGACGTAACCAT 114

L1450VP22012 ACAAAATGAACGTCTGCAAGAAAAAGAAA------TTGAAAATAATACAGACGTAACCAT 114

L1621VP22013 ACAAAATGAACGTCTGCAAGAAAAAGAAA------TTGAAAATAATACAGACGTAACCAT 114

R107VP22003 ACAAAATGAACGTCTGCAAGAAAAAGAAA------TTGAAAATAATACAGACGTGGCCAT 114

R303VP22004 ACAAAATGAACGTCTGCAAGAAAAAGAAA------TTGAAAATAATACAGACGTGGCCAT 114

R709VP22005 ACAAAATGAACGTCTGCAAGAAAAAGAAA------TTGAAAATAATACAGACGTAACCAT 114

R1267VP22006 ACAAAATGAACGTCTGCAAGAAAAAGAAA------TTGAAAATAATACAGACGTAACCAT 114

R1604VP22011 ACAAAATGAACGTCTGCAAGAAAAAGAAA------TTGAAAATAATACAGACGTAACCAT 114

Y106VP22004 ACAAAATGAACGTTTGCAAGAAAAAGAGA------TTGAAAATAATACAGACGTAACCAT 114

Y111VP22004 ACAAAATGAACGTCTGCAAGAAAAAGAAA------TTGAAAATAATACAGACGTAACTAT 114

Z1557VP22011 ACAAAATGAACGTCTGCAAGAAAAAGAAA------TTGAAAATAATACAGACGTAACCAT 114

Z1602VP22012 ACAAAATGAACGTCTGCAAGAAAAAGAAA------TTGAAAATAATACAGACGTAACCAT 114

************* ******* ***** * ****** ************ **

115 135

31VP22002 GGAAAATAAAAATAAAAATA------GAAATAATAATAGAAAGCAGCAATTATCTGACAA 168

723VP22003 GGAAAATAAAAATAAAAATA------GAAATAATAATAGAAAGCAGCAATTATCTGACAA 168

A16VP22000 GGAAAATAAAAATAAAAATA------GAAATAATAATAGAAAGCAGCAATTATCTGACAA 168

E093VP22007 GGAAAATAAAAATAAAAATA------GAAATAATAATAGAAAGCAGCAATTATCTGACAA 168

E329VP22007 GGAAAATAAGAGTAAAAATA------GAAATAATAATAGAAAGCAGCAATTATCTGACAA 168

E566VP22007 GGAAAATAAAAATAAAAATA------GAAATAATAATAGAAAGCAGCAATTATCTGACAA 168

E707VP22007 GGAAAATAAAAATAAAAATA------GAAATAATAATAGAAAGCAGCAATTATCTGACAA 168

E956VP22008 GGAAAATAAAAATAAAAATA------GAAATAATAATAGAAAGCAGCAATTATCTGACAA 168

E1367VP22008 GGAAAATAAAAATAAAAATA------GAAATAATAATAGAAAGCAGCAATTATCTGACAA 168

E1857VP22009 GGAAAATAAAAATAAAAATA------GAAATAATAATAGAAAGCAGCAATTATCTGACAA 168

E1861VP22009 GGAAAATAAAAATAAAAATA------GAAATAATAATAGAAAGCAGCAATTATCTGACAA 168

E2000VP22010 GGAAAATAAAAATAAAAATA------GAAATAATAATAGAAAGCAGCAATTATCTGACAA 168

E2421VP22010 GGAAAATAAAAATAAAAATA------GAAATAATAATAGAAAGCAGCAATTATCTGACAA 168

E2422VP22010 GGAAAATAAAAATAAAAATA------GAAATAATAATAGAAAGCAGCAATTATCTGACAA 168

E2432VP22010 GGAAAATAAAAATAAAAATA------GAAATAATAATAGAAAGCAGCAATTATCTGACAA 168

E2461VP22011 GGAAAATAAAAATAAAAATA------GAAATAATAATAGAAAGCAGCAATTATCTGACAA 168

E2835VP22011 GGAAAATAAAAATAAAAATA------GAAATAATAATAGAAAGCAGCAATTATCTGACAA 168

E3239VP22012 GGAAAATAAAAATAAAAATA------GAAATAATAATAGAAAGCAGCAATTATCTGACAA 168

L148VP22004 GGAAAATAAAAATAAAAATA------GAAATAATAATAGAAAGCAGCAATTATCTGACAA 168

L210VP22004 GGAAAATAAAAATAAAAATA------GAAATAATAATAGAAAGCAGCAATTATCTGACAA 174

L478VP22006 GGAAAATAAAAATAAAAATA------GAAATAATAATAGAAAGCAGCAATTATCTGACAA 168

L1066VP22009 GGAAAATAAAAATAAAAATA------GAAATAATAATAGAAAGCAGCAATTATCTGACAA 168

L1450VP22012 GGAAAATAAAAATAAAAATAAAAATAGAAATAATAATAGAAATCAGCAATTATCTGACAA 174

L1621VP22013 GGAAAATAAAAATAAAAGTA------GAAATAATAATAGAAAGCAGCAATTATCTGACAA 168

R107VP22003 GGAAAATAAAAATAAAAATA------GAAATAATAATAGAAAGCAGCAATTATCTGACAA 168

R303VP22004 GGAAAATAAAAATAAAAATA------GAAATAATAATAGAAAGCAGCAATTATCTGACAC 168

R709VP22005 GGAAAATAAAAATAAAAATA------GAAATAATAATAGAAAGCAGCAATTATCTGACAA 168

R1267VP22006 GGAAAATAAAAATAAAAATA------GAAATAATAATAGAAAGCAGCAATTATCTGACAA 168

R1604VP22011 GGAAAATAAAAATAAAAATAAAAATAGAAATAATAATAGAAAGCAGCAATTATCTGACAA 174

Y106VP22004 GGAAAATAAAAATAAAAATA------GAAATAATAATAGATAGCAGCAATTATCTGACAA 168

Y111VP22004 GGAAAATAAAAATAAAAATA------GAAATAATAATAGAAAGCAGCAATTATCTGACAA 168

Z1557VP22011 GGAAAATAAAAATAAAAATAAAAATAGAAATAATAATAGAAAGCAGCAATTATCTGACAA 174

Z1602VP22012 GGAAAATAAAAATAAAAATAAAAATAGAAATAATAATAGAAAGCAGCAATTATCTGACAA 174

********* * ***** ** ************** * ****************
