# Supplementary material for: Molecular Epidemiology and Genetic Evolution of the Whole Genome of G3P[8] Human Rotavirus in Wuhan, China, from 2000 through 2013
Source: PLoS One. 2014 Mar 27;9(3):e88850. doi: 10.1371/journal.pone.0088850 (PMC3967987; doi:10.1371/journal.pone.0088850)
Supplement: Figure S3 — Alignment of partial VP2 amino acid sequences of the G3 rotavirus strains. (DOC) [file pone.0088850.s003.doc]

**Fig.S3** Alignment of partial VP2 amino acid sequences of the G3 rotavirus strains. Amino acids in green or blue represent an inserted sequence. Dash denotes gap, asterisk below alignment shows consensusamino acid. Amino acid numbers are shown above and right.

　　　　　　　　　　　　24 39

A16VP2 MAYRKRGAKREDLPQQNERLQEKE--IENNTDVIMENKNKN--RNNNRKQQLSDKVLSQK 56

31VP2 MAYRKRGAKREDLPQQNERLQEKE--IENNTDVAMENKNKN--RNNNRKQQLSDKVLSQK 56

723VP2 MAYRKRGAKREDLPQQNERLQEKE--IENNTDVAMENKNKN--RNNNRKQQLSDKVLSQK 56

R107VP2 MAYRKRGAKREDLPQQNERLQEKE--IENNTDVAMENKNKN--RNNNRKQQLSDKVLSQK 56

R303VP2 MAYRKRGAKREDLPQQNERLQEKE--IENNTDVAMENKNKN--RNNNRKQQLSDTVLSQK 56

Y106VP2 MAYRKRGAKREDLPQQNERLQEKE--IENNTDVTMENKNKN--RNNNRKQQLSDKVLSQK 56

L210VP2 MAYRKRGAKREDLPQQNERLQEKEKEIENNTDVTMENKNKN--RNNNRKQQLSDKVLSQK 58

L478VP2 MAYRKRGAKREDLPQQNERLQEKE--IENNTDVTMENKNKN--RNNNRKQQLSDKVLSQK 56

R1267VP2 MAYRKRGAKREDLPQQNERLQEKE--IENNTDVTMENKNKN--RNNNRKQQLSDKVLSQK 56

E093VP2 MAYRKRGAKREDLPQQNERLQEKE--IENNTDVTMENKNKN--RNNNRKQQLSDKVLSQK 56

E329VP2 MAYRKRGAKREDLPQQNERLQEKE--IENNTDVTMENKNKN--RNNNRKQQLSDKVLSQK 56

E707VP2 MAYRKRGAKREDLPQQNERLQEKE--IENNTDVTMENKNKN--RNNNRKQQLSDKVLSQK 56

E956VP2 MAYRKRGAKREDLPQQNERLQEKE--IENNTDVTMENKNKN--RNNNRKQQLSDKVLSQK 56

E1367VP2 MAYRKRGAKREDLPQQNERLQEKE--IENNTDVTMENKNKN--RNNNRKQQLSDKVLSQK 56

L1066VP2 MAYRKRGAKREDLPQQNERLQEKE--IENNTDVTMENKNKN--RNNNRKQQLSDKVLSQK 56

E1857VP2 MAYRKRGAKREDLPRQNERLQEKE--IENNTDVTMENKNKN--RNNNRKQQLSDKVLSQK 56

E1861VP2 MAYRKRGAKREDLPQQNERLQEKE--IENNTDVTMENKNKN--RNNNRKQQLSDKVLSQK 56

E2000VP2 MAYRKRGAKREDLPQQNERLQEKE--IENNTDVTMENKNKN--RNNNRKQQLSDKVLSQK 56

E2421VP2 MAYRKRGAKREDLPQQNERLQEKE--IENNTDVTMENKNKN--RNNNRKQQLSDKVLSQK 56

E2422VP2 MAYRKRGAKREDLPQQNERLQEKE--IENNTDVTMENKNKN--RNNNRKQQLSDKVLSQK 56

E2432VP2 MAYRKRGAKREDLPQQNERLQEKE--IENNTDVTMENKNKN--RNNNRKQQLSDKVLSQK 56

R1604VP2 MAYRKRGAKHEDLPQQNERLQEKE--IENNTDVTMENKNKNKNRNNNRKQQLSDKVLSQK 58

E2461VP2 MAYRKRGAKREDLPQQNERLQEKE--IENNTDVTMENKNKN--RNNNRKQQLSDKVLSQK 56

Z1557VP2 MAYRKRGAKREDLPQQNERLQEKE--IENNTDVTMENKNKNKNRNNNRKQQLSDKVLSQK 58

E2835VP2 MAYRKRGAKREDLPQQNERLQEKE--IEINTDVTMENKNKN--RNNNRKQQLSDKVLSQK 56

L1450VP2 MAYRKRGAKREDLPQQNERLQEKE--IENNTDVTMENKNKNKNRNNNRNQQLSDKVLSQK 58

Z1602VP2 MAYRKRGAKREDLPQQNERLQEKE--IENNTDVTMENKNKNKNRNNNRKQQLSDKVLSQK 58

E3239VP2 MAYRKRGAKREDLPQQNERLQEKE--IENNTDVTMENKNKN--RNNNRKQQLSDKVLSQK 56

L1621VP2 MAYRKRGAKREDLPQQNERLQEKE--IENNTDVTMENKNKS--RNNNRKQQLSDKVLSQK 56

Y111VP2 MAYRKRGAKREDLPQQNERLQEKE--IENNTDVTMENKNKN--RNNNRKQQLSDKVLSQK 56

L148VP2 MAYRKRGAKREDLPQQNERLQEKE--IENNTDVAMENKNKN--RNNNRKQQLSDKVLSQK 56

R709VP2 MAYRKRGAKREDLPQQNERLQEKE--IENNTDVTMENKNKN--RNNNRKQQLSDKVLSQK 56

E566VP2 MAYRKRGAKREDLPQQNERLQEKE--IENNTDVTMENKNKN--RNNNRKQQLSDKVLSQK 56

********* **** ********* ** **** ****** ***** ***** *****
