# Supplementary material for: Molecular Epidemiology and Genetic Evolution of the Whole Genome of G3P[8] Human Rotavirus in Wuhan, China, from 2000 through 2013
Source: PLoS One. 2014 Mar 27;9(3):e88850. doi: 10.1371/journal.pone.0088850 (PMC3967987; doi:10.1371/journal.pone.0088850)
Supplement: Figure S4 — Alignment of the amino acid residues defining the neutralization domains (designated as 7-1a, 7-1b and 7-2) of VP7 between the G3 strain in RotaTeq and Chinese G3P[8] RVA strains detected in Wuhan from 2000 to 2013. Red indicates the residues different from those of RotaTeq. (DOC) [file pone.0088850.s004.doc]

| **Fig. S4** Alignment of the amino acid residues defining the neutralization domains (designated as 7-1a, 7-1b and 7-2) of VP7 between the G3 strain in RotaTeq™ and Chinese G3P[8] RVA strains detected in Wuhan from 2000 to 2013. Red indicates the residues different from those of RotaTeq™. | **G3-VP7** | | | | | | | | | | | | | | | | | | | | | | | | | | | | | | |
| --- | --- | --- | --- | --- | --- | --- | --- | --- | --- | --- | --- | --- | --- | --- | --- | --- | --- | --- | --- | --- | --- | --- | --- | --- | --- | --- | --- | --- | --- | --- | --- |
|  | **7-1a** | | | | | | | | | | | | | |  | **7-1b** | | | | | |  | **7-2** | | | | | | | | |
| **RotaTeq-WI78-9/G3P[5]** | **87** | **91** | **94** | **96** | **97** | **98** | **99** | **100** | **104** | **123** | **125** | **129** | **130** | **291** |  | **201** | **211** | **212** | **213** | **238** | **242** |  | **143** | **145** | **146** | **147** | **148** | **190** | **217** | **221** | **264** |
|  | **T** | **T** | **N** | **N** | **S** | **W** | **K** | **D** | **Q** | **D** | **A** | **V** | **D** | **K** |  | **Q** | **D** | **A** | **N** | **K** | **D** |  | **K** | **D** | **A** | **T** | **L** | **S** | **E** | **A** | **G** |
| **CHN/A16/2000/G3P[8]** | **T** | **T** | **N** | **N** | **S** | **W** | **K** | **D** | **Q** | **D** | **A** | **V** | **D** | **K** |  | **Q** | **D** | **T** | **N** | **N** | **N** |  | **K** | **D** | **A** | **T** | **L** | **S** | **E** | **A** | **G** |
| **CHN/31/2002/G3P[8]** | **T** | **T** | **N** | **N** | **S** | **W** | **K** | **D** | **Q** | **D** | **A** | **V** | **D** | **K** |  | **Q** | **D** | **T** | **N** | **N** | **N** |  | **K** | **D** | **A** | **T** | **L** | **S** | **E** | **A** | **G** |
| **CHN/723/2003/G3P[8]** | **T** | **T** | **N** | **N** | **S** | **W** | **K** | **D** | **Q** | **D** | **A** | **V** | **N** | **K** |  | **Q** | **D** | **T** | **N** | **N** | **N** |  | **K** | **D** | **A** | **T** | **L** | **S** | **E** | **A** | **G** |
| **CHN/R107/2003/G3P[8]** | **T** | **T** | **N** | **N** | **S** | **W** | **K** | **D** | **Q** | **D** | **A** | **V** | **D** | **K** |  | **Q** | **D** | **T** | **N** | **N** | **N** |  | **K** | **D** | **A** | **T** | **L** | **S** | **E** | **A** | **G** |
| **CHN/R303/2004/G3P[8]** | **T** | **T** | **N** | **N** | **S** | **W** | **K** | **D** | **Q** | **D** | **A** | **V** | **D** | **K** |  | **Q** | **D** | **T** | **N** | **N** | **N** |  | **K** | **D** | **A** | **T** | **L** | **S** | **E** | **A** | **G** |
| **CHN/Y106/2004/G3P[8]** | **T** | **T** | **N** | **N** | **S** | **W** | **K** | **D** | **Q** | **D** | **A** | **V** | **D** | **K** |  | **Q** | **D** | **T** | **N** | **N** | **N** |  | **K** | **D** | **A** | **T** | **L** | **S** | **E** | **A** | **G** |
| **CHN/Y111/2004/G3P[8]** | **T** | **T** | **N** | **N** | **S** | **W** | **K** | **D** | **Q** | **D** | **A** | **V** | **N** | **K** |  | **Q** | **D** | **T** | **N** | **N** | **N** |  | **K** | **D** | **A** | **T** | **L** | **S** | **E** | **A** | **G** |
| **CHN/L148/2004/G3P[8]** | **T** | **T** | **N** | **N** | **S** | **W** | **K** | **D** | **Q** | **D** | **A** | **V** | **N** | **K** |  | **Q** | **D** | **T** | **N** | **N** | **N** |  | **K** | **D** | **A** | **T** | **L** | **S** | **E** | **A** | **G** |
| **CHN/L210/2005/G3P[8]** | **T** | **T** | **N** | **N** | **S** | **W** | **K** | **D** | **Q** | **D** | **A** | **V** | **N** | **K** |  | **Q** | **D** | **T** | **N** | **N** | **N** |  | **K** | **D** | **A** | **T** | **L** | **S** | **E** | **A** | **G** |
| **CHN/R709/2005/G3P[8]** | **T** | **T** | **N** | **N** | **S** | **W** | **K** | **D** | **Q** | **D** | **A** | **V** | **D** | **K** |  | **Q** | **D** | **T** | **N** | **N** | **N** |  | **K** | **D** | **A** | **T** | **L** | **S** | **E** | **A** | **G** |
| **CHN/L478/2006/G3P[8]** | **T** | **T** | **N** | **N** | **S** | **W** | **K** | **D** | **Q** | **D** | **A** | **V** | **D** | **K** |  | **Q** | **D** | **T** | **N** | **N** | **N** |  | **K** | **D** | **A** | **T** | **L** | **S** | **E** | **A** | **G** |
| **CHN/R1267/2006/G3P[8]** | **T** | **T** | **N** | **N** | **S** | **W** | **K** | **D** | **Q** | **D** | **A** | **V** | **D** | **K** |  | **Q** | **D** | **T** | **N** | **N** | **N** |  | **K** | **D** | **A** | **T** | **L** | **S** | **E** | **A** | **G** |
| **CHN/E093/2007/G3P[8]** | **T** | **T** | **N** | **N** | **S** | **W** | **K** | **D** | **Q** | **D** | **A** | **V** | **D** | **K** |  | **Q** | **D** | **T** | **N** | **N** | **N** |  | **K** | **D** | **A** | **T** | **L** | **S** | **E** | **A** | **G** |
| **CHN/E329/2007/G3P[8]** | **T** | **T** | **N** | **N** | **S** | **W** | **K** | **D** | **Q** | **D** | **A** | **V** | **D** | **K** |  | **Q** | **D** | **T** | **N** | **N** | **N** |  | **K** | **D** | **A** | **T** | **L** | **S** | **E** | **A** | **G** |
| **CHN/E566/2007/G3P[8]** | **T** | **T** | **N** | **N** | **S** | **W** | **K** | **D** | **Q** | **D** | **A** | **V** | **D** | **K** |  | **Q** | **D** | **T** | **N** | **N** | **N** |  | **K** | **D** | **A** | **T** | **L** | **S** | **E** | **A** | **G** |
| **CHN/E707/2007/G3P[8]** | **T** | **T** | **N** | **N** | **S** | **W** | **K** | **D** | **Q** | **D** | **A** | **V** | **D** | **K** |  | **Q** | **D** | **T** | **N** | **N** | **N** |  | **K** | **D** | **A** | **T** | **L** | **S** | **E** | **A** | **G** |
| **CHN/E956/2008/G3P[8]** | **T** | **T** | **N** | **N** | **S** | **W** | **K** | **D** | **Q** | **D** | **A** | **V** | **D** | **K** |  | **Q** | **D** | **T** | **N** | **N** | **N** |  | **K** | **D** | **A** | **T** | **L** | **S** | **E** | **A** | **G** |
| **CHN/E1367/2008/G3P[8]** | **T** | **T** | **N** | **N** | **S** | **W** | **K** | **D** | **Q** | **D** | **A** | **V** | **D** | **K** |  | **Q** | **D** | **T** | **N** | **N** | **N** |  | **K** | **D** | **A** | **T** | **L** | **S** | **E** | **A** | **G** |
| **CHN/L1066/2009/G3P[8]** | **T** | **T** | **N** | **N** | **S** | **W** | **K** | **D** | **Q** | **D** | **A** | **V** | **D** | **K** |  | **Q** | **D** | **T** | **N** | **N** | **N** |  | **K** | **D** | **A** | **T** | **L** | **S** | **E** | **A** | **G** |
| **CHN/E1857/2009/G3P[8]** | **T** | **T** | **N** | **N** | **S** | **W** | **K** | **D** | **Q** | **D** | **A** | **V** | **D** | **K** |  | **Q** | **D** | **T** | **N** | **N** | **N** |  | **K** | **D** | **A** | **T** | **L** | **S** | **E** | **A** | **G** |
| **CHN/E1861/2009/G3P[8]** | **T** | **T** | **N** | **N** | **S** | **W** | **K** | **D** | **Q** | **D** | **A** | **V** | **D** | **K** |  | **Q** | **D** | **T** | **N** | **N** | **N** |  | **K** | **D** | **A** | **T** | **L** | **S** | **E** | **A** | **G** |
| **CHN/E2000/2010/G3P[8]** | **T** | **T** | **N** | **N** | **S** | **W** | **K** | **D** | **Q** | **D** | **A** | **V** | **D** | **K** |  | **Q** | **D** | **T** | **N** | **N** | **N** |  | **K** | **D** | **A** | **T** | **L** | **S** | **E** | **A** | **G** |
| **CHN/E2421/2010/G3P[8]** | **T** | **T** | **N** | **N** | **S** | **W** | **K** | **D** | **Q** | **D** | **A** | **V** | **D** | **K** |  | **Q** | **D** | **T** | **N** | **N** | **N** |  | **K** | **D** | **A** | **T** | **L** | **S** | **E** | **A** | **G** |
| **CHN/E2422/2010/G3P[8]** | **T** | **T** | **N** | **N** | **S** | **W** | **K** | **D** | **Q** | **D** | **A** | **V** | **D** | **K** |  | **Q** | **D** | **T** | **N** | **N** | **N** |  | **K** | **D** | **A** | **T** | **L** | **S** | **E** | **A** | **G** |
| **CHN/E2432/2010/G3P[8]** | **T** | **T** | **N** | **N** | **S** | **W** | **K** | **D** | **Q** | **D** | **A** | **V** | **D** | **K** |  | **Q** | **D** | **T** | **N** | **N** | **N** |  | **K** | **D** | **A** | **T** | **L** | **S** | **E** | **A** | **G** |
| **CHN/R1604/2011/G3P[8]** | **T** | **T** | **N** | **N** | **S** | **W** | **K** | **D** | **Q** | **D** | **A** | **V** | **D** | **K** |  | **Q** | **D** | **T** | **N** | **N** | **N** |  | **K** | **D** | **A** | **T** | **L** | **S** | **E** | **A** | **G** |
| **CHN/E2461/2011/G3P[8]** | **T** | **T** | **N** | **N** | **S** | **W** | **K** | **D** | **Q** | **D** | **A** | **V** | **D** | **K** |  | **Q** | **D** | **T** | **N** | **N** | **N** |  | **K** | **D** | **A** | **T** | **L** | **S** | **E** | **A** | **G** |
| **CHN/Z1557/2011/G3P[8]** | **T** | **T** | **N** | **N** | **S** | **W** | **K** | **D** | **Q** | **D** | **A** | **V** | **D** | **K** |  | **Q** | **D** | **T** | **N** | **N** | **N** |  | **K** | **D** | **A** | **T** | **L** | **S** | **E** | **A** | **G** |
| **CHN/E2835/2011/G3P[8]** | **T** | **T** | **N** | **N** | **S** | **W** | **K** | **D** | **Q** | **D** | **A** | **V** | **D** | **K** |  | **Q** | **D** | **T** | **N** | **N** | **N** |  | **K** | **D** | **A** | **T** | **L** | **S** | **E** | **A** | **G** |
| **CHN/Z1602/2012/G3P[8]** | **T** | **T** | **N** | **N** | **S** | **W** | **K** | **D** | **Q** | **D** | **A** | **V** | **D** | **K** |  | **Q** | **D** | **T** | **N** | **N** | **N** |  | **K** | **D** | **A** | **T** | **L** | **S** | **E** | **A** | **G** |
| **CHN/L1450/2012/G3P[8]** | **T** | **T** | **N** | **N** | **S** | **W** | **K** | **D** | **Q** | **D** | **A** | **V** | **D** | **K** |  | **Q** | **D** | **T** | **N** | **N** | **N** |  | **K** | **D** | **A** | **T** | **L** | **S** | **E** | **A** | **G** |
| **CHN/E3239/2012/G3P[8]** | **T** | **T** | **N** | **N** | **S** | **W** | **K** | **D** | **Q** | **D** | **A** | **V** | **D** | **K** |  | **Q** | **D** | **T** | **N** | **N** | **N** |  | **K** | **D** | **A** | **T** | **L** | **S** | **E** | **A** | **G** |
| **CHN/L1621/2013/G3P[8]** | **T** | **T** | **N** | **N** | **S** | **W** | **K** | **D** | **Q** | **D** | **A** | **V** | **D** | **K** |  | **Q** | **D** | **T** | **N** | **N** | **N** |  | **K** | **D** | **A** | **T** | **L** | **S** | **E** | **A** | **G** |
