# Supplementary material for: Molecular Epidemiology and Genetic Evolution of the Whole Genome of G3P[8] Human Rotavirus in Wuhan, China, from 2000 through 2013
Source: PLoS One. 2014 Mar 27;9(3):e88850. doi: 10.1371/journal.pone.0088850 (PMC3967987; doi:10.1371/journal.pone.0088850)
Supplement: Figure S6 — Alignment of the amino acid residues corresponding to those defining the VP4 neutralization domains designated as 5-1, 5-2, 5-3, 5-4 and 5-5 in the VP5* subunit between the P[8] strains in Rotarix and RotaTeq and Chinese G3P[8] RVA strains detected in Wuhan from 2000 to 2013. (DOC) [file pone.0088850.s006.doc]

**Fig. S6**Alignment of the amino acid residues corresponding to those defining the VP4 neutralization domains designated as 5-1, 5-2, 5-3, 5-4 and 5-5 in the VP5* subunit between the P[8] strains in Rotarix™ and RotaTeq™ and Chinese G3P[8] RVA strains detected in Wuhan from 2000 to 2013. Green indicates the residues different from those of both Rotarix™ and RotaTeq™. Red indicates the residues identical to those of RotaTeq™, but different from those of Rotarix™. Blue indicates the residues identical to those of Rotarix™, but different from those of RotaTeq™.

|  | **P[8]-VP5*** | | | | | | | | | | | | | | | |
| --- | --- | --- | --- | --- | --- | --- | --- | --- | --- | --- | --- | --- | --- | --- | --- | --- |
|  | **5-1** | | | | | | | |  | **5-2** |  | **5-3** |  | **5-4** |  | **5-5** |
|  | **384** | **386** | **388** | **393** | **394** | **398** | **440** | **441** |  | **434** |  | **459** |  | **429** |  | **306** |
| **Rotarix-A41CB052A/G1P[8]** | **S** | **Y** | **S** | **A** | **W** | **N** | **L** | **R** |  | **E** |  | **N** |  | **S** |  | **L** |
| **RotaTeq-WI79-4/G6P1A[8]** | **R** | **H** | **S** | **A** | **W** | **N** | **L** | **R** |  | **E** |  | **N** |  | **S** |  | **L** |
| **CHN/A16/2000/G3P[8]** | **S** | **D** | **S** | **A** | **W** | **N** | **L** | **R** |  | **E** |  | **N** |  | **S** |  | **L** |
| **CHN/31/2002/G3P[8]** | **S** | **D** | **S** | **A** | **W** | **N** | **L** | **R** |  | **E** |  | **N** |  | **S** |  | **L** |
| **CHN/723/2003/G3P[8]** | **S** | **D** | **S** | **A** | **W** | **N** | **L** | **R** |  | **E** |  | **N** |  | **S** |  | **L** |
| **CHN/R107/2003/G3P[8]** | **S** | **D** | **S** | **A** | **W** | **N** | **L** | **R** |  | **E** |  | **N** |  | **S** |  | **L** |
| **CHN/R303/2004/G3P[8]** | **S** | **D** | **S** | **T** | **W** | **N** | **L** | **R** |  | **E** |  | **N** |  | **S** |  | **L** |
| **CHN/Y106/2004/G3P[8]** | **S** | **D** | **S** | **A** | **W** | **N** | **L** | **R** |  | **E** |  | **N** |  | **S** |  | **L** |
| **CHN/Y111/2004/G3P[8]** | **S** | **D** | **S** | **A** | **W** | **N** | **L** | **R** |  | **E** |  | **N** |  | **S** |  | **L** |
| **CHN/L148/2004/G3P[8]** | **S** | **D** | **S** | **A** | **W** | **N** | **L** | **R** |  | **E** |  | **N** |  | **S** |  | **L** |
| **CHN/L210/2005/G3P[8]** | **S** | **D** | **S** | **A** | **W** | **N** | **L** | **R** |  | **E** |  | **N** |  | **S** |  | **L** |
| **CHN/R709/2005/G3P[8]** | **S** | **D** | **S** | **A** | **W** | **N** | **L** | **R** |  | **E** |  | **N** |  | **S** |  | **L** |
| **CHN/L478/2006/G3P[8]** | **S** | **D** | **S** | **A** | **W** | **N** | **L** | **R** |  | **E** |  | **N** |  | **S** |  | **L** |
| **CHN/R1267/2006/G3P[8]** | **S** | **D** | **S** | **A** | **W** | **N** | **L** | **R** |  | **E** |  | **N** |  | **S** |  | **L** |
| **CHN/E093/2007/G3P[8]** | **S** | **D** | **S** | **A** | **W** | **N** | **L** | **R** |  | **E** |  | **N** |  | **S** |  | **L** |
| **CHN/E329/2007/G3P[8]** | **S** | **D** | **S** | **A** | **W** | **N** | **L** | **R** |  | **E** |  | **N** |  | **S** |  | **L** |
| **CHN/E566/2007/G3P[8]** | **S** | **D** | **S** | **A** | **W** | **N** | **L** | **R** |  | **E** |  | **N** |  | **S** |  | **L** |
| **CHN/E707/2007/G3P[8]** | **S** | **D** | **S** | **A** | **W** | **N** | **L** | **R** |  | **E** |  | **N** |  | **S** |  | **L** |
| **CHN/E956/2008/G3P[8]** | **S** | **D** | **S** | **A** | **W** | **N** | **L** | **R** |  | **E** |  | **N** |  | **S** |  | **L** |
| **CHN/E1367/2008/G3P[8]** | **S** | **D** | **S** | **A** | **W** | **N** | **L** | **R** |  | **E** |  | **N** |  | **S** |  | **L** |
| **CHN/L1066/2009/G3P[8]** | **S** | **D** | **S** | **A** | **W** | **N** | **L** | **R** |  | **E** |  | **N** |  | **S** |  | **L** |
| **CHN/E1857/2009/G3P[8]** | **S** | **D** | **S** | **A** | **W** | **N** | **L** | **R** |  | **E** |  | **N** |  | **S** |  | **L** |
| **CHN/E1861/2009/G3P[8]** | **S** | **D** | **S** | **A** | **W** | **N** | **L** | **R** |  | **E** |  | **N** |  | **S** |  | **L** |
| **CHN/E2000/2010/G3P[8]** | **S** | **D** | **S** | **A** | **W** | **N** | **L** | **R** |  | **E** |  | **N** |  | **S** |  | **L** |
| **CHN/E2421/2010/G3P[8]** | **S** | **D** | **S** | **A** | **W** | **N** | **L** | **R** |  | **E** |  | **N** |  | **S** |  | **L** |
| **CHN/E2422/2010/G3P[8]** | **S** | **D** | **S** | **A** | **W** | **N** | **L** | **R** |  | **E** |  | **N** |  | **S** |  | **L** |
| **CHN/E2432/2010/G3P[8]** | **S** | **D** | **S** | **A** | **W** | **N** | **L** | **R** |  | **E** |  | **N** |  | **S** |  | **L** |
| **CHN/R1604/2011/G3P[8]** | **S** | **D** | **S** | **A** | **W** | **N** | **L** | **R** |  | **E** |  | **N** |  | **S** |  | **L** |
| **CHN/E2461/2011/G3P[8]** | **S** | **D** | **S** | **A** | **W** | **N** | **L** | **R** |  | **E** |  | **N** |  | **S** |  | **L** |
| **CHN/Z1557/2011/G3P[8]** | **S** | **D** | **S** | **A** | **W** | **N** | **L** | **R** |  | **E** |  | **N** |  | **S** |  | **L** |
| **CHN/E2835/2011/G3P[8]** | **S** | **D** | **S** | **A** | **W** | **N** | **L** | **R** |  | **E** |  | **N** |  | **S** |  | **L** |
| **CHN/Z1602/2012/G3P[8]** | **S** | **D** | **S** | **A** | **W** | **N** | **L** | **R** |  | **E** |  | **N** |  | **S** |  | **L** |
| **CHN/L1450/2012/G3P[8]** | **S** | **D** | **S** | **A** | **W** | **N** | **L** | **R** |  | **E** |  | **N** |  | **S** |  | **L** |
| **CHN/E3239/2012/G3P[8]** | **S** | **D** | **S** | **A** | **W** | **N** | **L** | **R** |  | **E** |  | **N** |  | **S** |  | **L** |
| **CHN/L1621/2013/G3P[8]** | **S** | **D** | **S** | **A** | **W** | **N** | **L** | **R** |  | **E** |  | **N** |  | **S** |  | **L** |
