# Supplementary material for: Molecular Epidemiology and Genetic Evolution of the Whole Genome of G3P[8] Human Rotavirus in Wuhan, China, from 2000 through 2013
Source: PLoS One. 2014 Mar 27;9(3):e88850. doi: 10.1371/journal.pone.0088850 (PMC3967987; doi:10.1371/journal.pone.0088850)
Supplement: Table S2 — Primers used for amplification of different genes of G3P[8] RVA strains. (DOC) [file pone.0088850.s008.doc]

**Table S2** Primers used for amplification of different genes of G3P[8] group A rotavirus.

| Gene | Primer | Sequence (5’-3’) | Polarity | Nucleotide Position | publication |
| --- | --- | --- | --- | --- | --- |
| VP1 | VP1-1F | GGC TAT TAA AGC TGT ACA ATG G | + | 1-22 | This study |
|  | VP1-795R | ACCATTGATATCTATTAATG | - | 795-776 | This study |
|  | VP1- 524F | TATTTACCATAGTGGCATCTAC | + | 524-545 | This study |
|  | VP1-1650R | CAT ATC TAA ACC CAT AAT TAT TCC | - | 1650-1627 | This study |
|  | VP1-1375F | GATGTTCCTGGACGAAGAAC | + | 1375-1394 | This study |
|  | VP1-2294R | GTAGTTAACACTCGTTCTGACGG | - | 2294-2272 | This study |
|  | VP1-1903F | GATGGTGATGATAATTATGC | + | 1903-1922 | This study |
|  | VP1-2645R | TCATTAATAGTAATTTTATTT | - | 2645-2625 | This study |
|  | VP1-2492F | TAATTGTATCCAAAGGTATAG | + | 2492-2512 | This study |
|  | VP1-3302R | GGT CAC ATC TAA GCGCTC | - | 3302-3285 | This study |
| VP2 | VP2-1F | GGC TAT TAA AGG CTC AAT GG | + | 1-20 | This study |
|  | VP2-854R | CGTTATTTAATGGTTCAACT | - | 854-835 | This study |
|  | VP2-643F | TAAAGTTGTAGATTCTGAGA | + | 643-662 | This study |
|  | VP2-1422R | GGAGTCTGAGGATCGCCATT | - | 1422-1403 | This study |
|  | VP2-1380F | GAATGCAAAGAATGCATTATAG | + | 1380-1401 | This study |
|  | VP2-2027R | CTCGTGGTACATCAAAAATT | - | 2027-2008 | This study |
|  | VP2-1931F | ATTACGGCTGCTAATAGACT | + | 1931-1950 | This study |
|  | VP2-2729R | GGT CAT ATC TCC ACA GTG G | - | 2729-2711 | This study |
| VP3 | VP3-1F | GGCTATTAAAGCAGTACTAGT | + | 1-21 | This study |
|  | VP3-896R | ATATTATTGGTTTCGAATGG | - | 896-877 | This study |
|  | VP3-815F | ATTGGACAATATAAAAATATG | + | 815-835 | This study |
|  | VP3-1585R | GGCAGAAACGTCCAATCAT | - | 1585-1567 | This study |
|  | VP3-1364F | ACA GAG AAT GTG TTT ATA C | + | 1364-1382 | This study |
|  | VP3-2133R | TCA GCA TAY GTT ATA CTA TAT AC | - | 2133-2111 | This study |
|  | VP3-1888F | TTATAATGCATTGATTTACT | + | 1888-1907 | This study |
|  | VP3 -2591R | GGTCACATCATGACTAGTGTG | - | 2591-2571 | This study |
| VP4 | VP4-1F | GGC TAT AAA ATG GCT TCG CTC A | + | 1-22 | This study |
|  | VP4-1185R | GAC TGG CCA TGC ACC TAC AGG T | - | 1185-1164 | This study |
|  | VP4-788F | TATGGAAAGAAATGCAATAT | + | 788-807 | This study |
|  | VP4-1625R | AGATCAATTGTGCTTTTAAT | - | 1625-1606 | This study |
|  | VP4-1515F | GGAATTYAATTCATTATCACAAG | + | 1515-1537 | This study |
|  | VP4-R | GGTCACATCCTCAATAGC | - | 2359-2342 | Mc. Donald et al., 2009 |
| VP6 | VP6-1F | GGC TTT TWA AAC GAA GTC TTC | + | 1-21 | Mc. Donald et al., 2009 |
|  | VP6 -856R | CCAAATCTAGCTTGATATGT | - | 856-837 | This study |
|  | VP6 -711F | GAAAGATTYAGTTTTCCAAGAGT | + | 711-733 | Mc. Donald et al., 2009 |
|  | VP6R | GGT CAC ATC CTC TCA CT | - | 1356-1340 | Mc. Donald et al., 2009 |
| VP7 | C2 | GGC TTT AAA AGA GAG AAT TTC CGT CTG G | + | 1-28 | Taniguchi et al., 1992 |
|  | C1 | GGT CAC ATC ATA CAA TTC TAA TCT AAG | - | 1062-1039 | Taniguchi et al., 1992 |
| NSP1 | NSP1-1F | TTT ATG AAA AGT CTT GTG | + | 8-25 | Mc. Donald et al., 2009 |
|  | NSP1-869R | ATTCRATATGTGATGTTTT | - | 869-851 | This study |
|  | NSP1-531F | TTAATCAAACTCCATTCTC | + | 531-549 | This study |
|  | NSP1R | GGT CAC ATT TTA TGC TGC CTA G | - | 1566-1545 | This study |
| NSP2 | NSP2F | GGC TTT TAA MGC GTC TCA GTC | + | 1-21 | Mc. Donald et al., 2009 |
|  | NSP2R | GGT CAC ATA AGC GCT TTC | - | 1059-1042 | Mc. Donald et al., 2009 |
| NSP3 | NSP3F | GGC TTT TAA TGC TTT TCA GTG G | + | 1-22 | Mc. Donald et al., 2009 |
|  | NSP3R | GGT CAC ATA ACG CCC CTA TAG | - | 1074-1054 | This study |
| NSP4 | NSP4F | GGC TTT TAA AAG TTC TGT T | + | 1-19 | This study |
|  | NSP4R | GGT CAC RYT AAG ACC RTT CC | - | 750-731 | Mc. Donald et al., 2009 |
| NSP5 | NSP5F | GGC TTT WAA AGC GCT ACA GTG | + | 1-21 | Mc. Donald et al., 2009 |
|  | NSP5R | GGT CAC AAA ACG GGA GTG | - | 664-647 | This study |

More than one nucleotide base at a primer position is denoted by upper case letter R (A or G), Y (C or T), M (A or C), K (G or T), S (G or C), W (A or T), H (A, T or C), B (G, T or C), V (G, A or C), D (G, A or T) or N (A, T, G or C)

**References:**

McDonald, S.M., Matthijnssens, J., McAllen, J.K., Hine, E., Overton, L., Wang, S., Lemey, P., Zeller, M., Van Ranst, M., Spiro, D.J., Patton, J.T., 2009. Evolutionary dynamics of human rotaviruses: balancing reassortment with preferred genome constellations. PLoS. Pathog. 5:e1000634.

Taniguchi, K., Wakasugi, F., Pongsuwanna, Y., Urasawa, T., Ukae, S., Chiba, S., Urasawa, S., 1992. Identification of human and bovine rotavirus serotypes by polymerase chain reaction. Epidemiol. Infect. 109, 303-312.
