# Supplementary material for: Molecular Epidemiology and Genetic Evolution of the Whole Genome of G3P[8] Human Rotavirus in Wuhan, China, from 2000 through 2013
Source: PLoS One. 2014 Mar 27;9(3):e88850. doi: 10.1371/journal.pone.0088850 (PMC3967987; doi:10.1371/journal.pone.0088850)
Supplement: Table S3 — Amino acid differences among the Chinese G3P[8] RVA strains belonging to different phylogenetic lineages. (DOC) [file pone.0088850.s009.doc]

**Table S3** Amino acid (aa) differences among the Chinese G3P[8] RVA strains belonging to different phylogenetic lineages (indicated by various colors in Fig. 1). Amino acid changes limited to one or a few RVA strains are underlined. Amino acid changes limited to only one or two RVA strains within the major lineage (green) are not shown.

|  |  | Phylogenetic lineage | | | | |  |
| --- | --- | --- | --- | --- | --- | --- | --- |
| Protein | aa no. | Green | Orange | Red | Blue | Grey |  |
| VP1 | 47 | S | N | S | S | S |  |
|  | 52 | G/S/D | G | G | S | G |  |
|  | 53 | M/I | I | M | L | M |  |
|  | 67 | D | D | N | D | D |  |
|  | 90 | V | V | V/A | V | V |  |
|  | 159 | A | A | A/T | A | A |  |
|  | 293 | D | D | D | D | E |  |
|  | 296 | K | K | K | R | K |  |
|  | 305 | R | R | R | K | R |  |
|  | 352 | T | T | T | T | I |  |
|  | 357 | G/D/S | G | G | G | G |  |
|  | 372 | V/I | V | V | V | V |  |
|  | 408 | L | I | L | L | L |  |
|  | 411 | G | G | G/S | G | G |  |
|  | 415 | I | I | V | I | I |  |
|  | 432 | R | K | R | R | R |  |
|  | 443 | V | V | V | I | V |  |
|  | 486 | H | H | H/N | H | H |  |
|  | 544 | M | M | M | I | M |  |
|  | 545 | L | L | L | Q | L |  |
|  | 555 | I | I | I | V | I |  |
|  | 563 | Q | Q | Q/R | Q | Q |  |
|  | 580 | V | V | V | I | V |  |
|  | 657 | H/R | H | Y | Y | H |  |
|  | 821 | I | I | I | V | I |  |
|  | 825 | V | V | V | L | V |  |
|  | 886 | V/I | V | V | I | V |  |
|  | 898 | K | K | K/R | K | K |  |
|  | 924 | S | S | S | T | S |  |
|  | 973 | G | G/S | G | G | G |  |
|  | 999 | I | I | I | I | V |  |
|  | 1044 | H/Y | H | Y | Y | H |  |
|  |  |  |  |  |  |  |  |
|  |  |  |  |  |  |  |  |
| Protein | aa no. | Green | Orange | Cyan |  |  |  |
| VP4 | 7 | R | R/K | R |  |  |  |
|  | 19 | Y | Y | H |  |  |  |
|  | 30 | T/A | T | T |  |  |  |
|  | 63 | P | P | P/S |  |  |  |
|  | 78 | S | T | T |  |  |  |
|  | 91 | V | V/I | V |  |  |  |
|  | 92 | V | V | I |  |  |  |
|  | 113 | D | D | N |  |  |  |
|  | 114 | P | P | S |  |  |  |
|  | 147 | S | S | N |  |  |  |
|  | 149 | N | S/N | N |  |  |  |
|  | 162 | R | K/R | R |  |  |  |
|  | 173 | I | I | V |  |  |  |
|  | 242 | I | I | V |  |  |  |
|  | 245 | K | T | K |  |  |  |
|  | 256 | S | S | S/P |  |  |  |
|  | 264 | M | M | M/V |  |  |  |
|  | 338 | I | I | V |  |  |  |
|  | 545 | T | T/A | T |  |  |  |
|  | 565 | N | N/T | N |  |  |  |
|  | 577 | S | S/N | S |  |  |  |
|  | 580 | V/I | V | I |  |  |  |
|  | 587 | V | F | V |  |  |  |
|  | 608 | A | S | S |  |  |  |
|  | 621 | K | R/K | K |  |  |  |
|  | 674 | I | I/V | I |  |  |  |
|  | 697 | L | L/F | L |  |  |  |
|  | 708 | A | A | T |  |  |  |
|  | 747 | N | N | D |  |  |  |
|  |  |  |  |  |  |  |  |
|  |  |  |  |  |  |  |  |
| Protein | aa no. | Green | Orange | Cyan | Pink |  |  |
| VP6 | 66 | I | I | V | I |  |  |
|  | 80 | T | T | N | T |  |  |
|  | 120 | A | A | S | A |  |  |
|  | 134 | E | E | K | E |  |  |
|  | 199 | L | L | I | L |  |  |
|  | 252 | I | I | V | I |  |  |
|  | 281 | V | V | I | V |  |  |
|  |  |  |  |  |  |  |  |
|  |  |  |  |  |  |  |  |
| Protein | aa no. | Green | Orange | Red | Pink |  |  |
| NSP1 | 7 | A | A | V | A |  |  |
|  | 10 | H | Y | Y | Y |  |  |
|  | 19 | N | H | N | H |  |  |
|  | 20 | T | A | T | S |  |  |
|  | 29 | T | T/I | T | T |  |  |
|  | 32 | P | P | P | T |  |  |
|  | 38 | C | Y | Y | Y |  |  |
|  | 49 | T/A | T | T | T |  |  |
|  | 55 | S | R | S | R |  |  |
|  | 59 | M | M | I | M |  |  |
|  | 67 | N | S | S | S |  |  |
|  | 68 | Q | Q/R | Q | Q |  |  |
|  | 70 | G | D | G | D |  |  |
|  | 77 | E | Q | E | Q |  |  |
|  | 93 | D | N | D | N |  |  |
|  | 96 | K/N | M | K | M |  |  |
|  | 100 | D/N/G | D | D | D |  |  |
|  | 103 | E | N | E | N |  |  |
|  | 104 | T | I | I | I |  |  |
|  | 108 | I | I | M | I |  |  |
|  | 110 | H | Q | H | Q |  |  |
|  | 111 | K | R | K | R |  |  |
|  | 114 | C | D | R | D |  |  |
|  | 115 | R | K | R | K |  |  |
|  | 118 | N | S | N | N |  |  |
|  | 119 | N | S | T | S |  |  |
|  | 157 | I | V | V | V |  |  |
|  | 163 | N | S | D | S |  |  |
|  | 165 | N/D | R | N | R |  |  |
|  | 166 | N | D | N | D |  |  |
|  | 167 | I | V | I | I |  |  |
|  | 175 | V | T | I | T |  |  |
|  | 178 | V | I | V | I |  |  |
|  | 180 | I | M/V | I | M |  |  |
|  | 188 | D/H | N | D | N |  |  |
|  | 192 | T | N | T | N |  |  |
|  | 199 | A | V | A | V |  |  |
|  | 200 | S | A | S | A |  |  |
|  | 207 | S | S | N | S |  |  |
|  | 209 | Q | K | Q | K |  |  |
|  | 213 | V | I | I | I |  |  |
|  | 214 | N | S | N | S |  |  |
|  | 216 | S | L | S | L |  |  |
|  | 220 | I | T | I | T |  |  |
|  | 223 | L | I | L | I |  |  |
|  | 246 | I | T | I | T |  |  |
|  | 253 | S | S/F | S | P |  |  |
|  | 254 | F | S | F | S |  |  |
|  | 255 | D/E/N | D | D | D |  |  |
|  | 258 | E/K/G | G | E | G |  |  |
|  | 265 | N | D | N | D |  |  |
|  | 266 | V | M | V | M |  |  |
|  | 267 | S | S/N | S | S |  |  |
|  | 268 | A | T | A | T |  |  |
|  | 271 | D | N | D | N |  |  |
|  | 272 | M | V | M | V |  |  |
|  | 275 | T | T/K | T | T |  |  |
|  | 283 | V | I | V | I |  |  |
|  | 285 | T/S | S | S | S |  |  |
|  | 289 | I | M | I | I |  |  |
|  | 292 | Q | Q | H | Q |  |  |
|  | 293 | H | Y | C | H |  |  |
|  | 297 | M | I | M | I |  |  |
|  | 301 | L | H | L | H |  |  |
|  | 307 | I/V | V | I | V |  |  |
|  | 312 | K | R | K | K |  |  |
|  | 314 | L | S/A | L | S |  |  |
|  | 319 | H/R | H | H | H |  |  |
|  | 326 | V | I | I | I |  |  |
|  | 329 | N | G | S | G |  |  |
|  | 330 | Y | Y/H | Y | Y |  |  |
|  | 336 | F | F/V | F | F |  |  |
|  | 357 | V | S | V | S |  |  |
|  | 371 | Y | H | Y | H |  |  |
|  | 372 | V | V | V | I |  |  |
|  | 373 | E | K | K | K |  |  |
|  | 374 | D/N | N | D | D |  |  |
|  | 377 | N | D | N | D |  |  |
|  | 378 | V | V | V | I |  |  |
|  | 381 | D | D/N | D | D |  |  |
|  | 382 | E/V | E | E | E |  |  |
|  | 383 | R | K | R | K |  |  |
|  | 386 | T | M/I | T | M |  |  |
|  | 387 | S | A | S | A |  |  |
|  | 388 | I | V | I | V |  |  |
|  | 389 | M | A | T | A |  |  |
|  | 390 | K | E | E | E |  |  |
|  | 398 | V | V/I | V | V |  |  |
|  | 402 | D | T | D | T |  |  |
|  | 403 | G | A | V | V |  |  |
|  | 408 | V/F/I | F | F | F |  |  |
|  | 412 | V/I | V | I | V |  |  |
|  | 419 | V | L | V | L |  |  |
|  | 421 | V | V | I | V |  |  |
|  | 422 | H | Q | H | Q |  |  |
|  | 424 | I | V | I | V |  |  |
|  | 428 | P | P | P | S |  |  |
|  | 434 | D | N | E | N |  |  |
|  | 435 | N | D | N | D |  |  |
|  | 436 | V | I | V | I |  |  |
|  | 437 | I | I | I | V |  |  |
|  | 438 | T | I | A | I |  |  |
|  | 441 | Q | K | Q | K |  |  |
|  | 450 | L | I | V | I |  |  |
|  | 459 | V | T | V | T |  |  |
|  | 463 | V | V | I | V |  |  |
|  | 470 | Y | C | Y | C |  |  |
|  | 473 | L | V | L | V |  |  |
|  | 477 | E | D | E | D |  |  |
|  | 485 | I | I | I | V |  |  |
|  |  |  |  |  |  |  |  |
|  |  |  |  |  |  |  |  |
| Protein | aa no. | Green | Orange | Blue | Pink |  |  |
| NSP2 | 23 | N | N | S | S |  |  |
|  | 47 | V | V | I | I |  |  |
|  | 57 | R | R | K | K |  |  |
|  | 63 | S | S | N | N |  |  |
|  | 74 | I | I | L | P |  |  |
|  | 96 | E | E | D | D |  |  |
|  | 97 | V | V | V | I |  |  |
|  | 135 | S | S | A | A |  |  |
|  | 158 | V | V | V | T |  |  |
|  | 197 | I | I | I | L |  |  |
|  | 200 | V | V | I | L |  |  |
|  | 202 | V | V | I | V |  |  |
|  | 205 | L | I | L | L |  |  |
|  | 218 | I | V | V | V |  |  |
|  | 229 | V | V | I | V |  |  |
|  | 249 | A | A | A | V |  |  |
|  | 251 | S/N | S | S | S |  |  |
|  | 254 | T | T | V | G |  |  |
|  | 255 | T | N | I | T |  |  |
|  | 282 | I | I | L | L |  |  |
|  | 284 | V | V | V | I |  |  |
|  |  |  |  |  |  |  |  |
|  |  |  |  |  |  |  |  |
| Protein | aa no. | Green | Orange | Pink |  |  |  |
| NSP3 | 9 | S | S | G |  |  |  |
|  | 65 | T | T | I |  |  |  |
|  | 76 | G | G/S | G |  |  |  |
|  | 78 | A/P | A | V |  |  |  |
|  | 79 | I | I | M |  |  |  |
|  | 104 | T | T | M |  |  |  |
|  | 138 | R | K | R |  |  |  |
|  | 141 | K | K | R |  |  |  |
|  | 155 | Y | Y | F |  |  |  |
|  | 156 | V | I | V |  |  |  |
|  | 169 | S | S/F | S |  |  |  |
|  | 185 | V | V/I | V |  |  |  |
|  | 186 | T | T | S |  |  |  |
|  | 191 | T | T | N |  |  |  |
|  | 204 | Y | Y | H |  |  |  |
|  | 229 | A | T | T |  |  |  |
|  | 233 | G | G | N |  |  |  |
|  | 235 | F | F | V |  |  |  |
|  | 255 | V | V | I |  |  |  |
|  | 257 | N | N | T |  |  |  |
|  | 268 | L | L | A |  |  |  |
|  | 271 | S/P | P | P |  |  |  |
|  | 272 | I | I | T |  |  |  |
|  | 275 | I | I | V |  |  |  |
|  | 278 | I | I | L |  |  |  |
|  | 282 | I | V | I |  |  |  |
|  | 286 | I | V | I |  |  |  |
|  | 297 | K | K | R |  |  |  |
|  | 300 | L | L | V |  |  |  |
|  | 301 | K | K | R |  |  |  |
|  | 307 | Y | Y | C |  |  |  |
|  | 308 | A | V | T |  |  |  |
|  | 309 | C | Y | Y |  |  |  |
|  |  |  |  |  |  |  |  |
|  |  |  |  |  |  |  |  |
| Protein | aa no. | Green | Orange |  |  |  |  |
| NSP4 | 12 | S | S/N |  |  |  |  |
|  | 76 | V | I |  |  |  |  |
|  | 141 | T | I |  |  |  |  |
|  | 142 | I | V/I |  |  |  |  |
|  | 153 | I | V |  |  |  |  |
|  |  |  |  |  |  |  |  |
| Protein | aa no. | Green | Orange |  |  |  |  |
| NSP5 | 16 | S | S/N |  |  |  |  |
|  | 116 | M/I | I |  |  |  |  |
|  | 138 | K | K/N |  |  |  |  |
